# Gut instinct: harnessing the power of probiotics to tame pathogenic signaling pathways in ulcerative colitis

**DOI:** 10.3389/fmed.2024.1396789

**Published:** 2024-09-11

**Authors:** Chou-Yi Hsu, Mohammed Ahmed Mustafa, Thabit Moath Omar, Sada Gh Taher, Mohammed Ubaid, Nataliya S. Gilmanova, Mustafa Nasrat Abdulraheem, Mohamed J. Saadh, Aya H. Athab, Rasoul Mirzaei, Sajad Karampoor

**Affiliations:** ^1^Department of Pharmacy, Chia Nan University of Pharmacy and Science, Tainan, Taiwan; ^2^Thunderbird School of Global Management, Arizona State University Tempe Campus, Phoenix, AZ, United States; ^3^Department of Medical Laboratory Technology, Imam Jaafar AL-Sadiq University, Baghdad, Iraq; ^4^Department of Pathological Analyzes, College of Applied Sciences, University of Samarra, Samarra, Iraq; ^5^Department of Medical Laboratory Technics, College of Health and Medical Technology, Alnoor University, Mosul, Iraq; ^6^Department of Pharmacy, National University of Science and Technology, Dhi Qar, Iraq; ^7^Department of MTL, Medical Technical College, Al-Farahidi University, Baghdad, Iraq; ^8^Department of Prosthetic Dentistry, I.M. Sechenov First Moscow State Medical University (Sechenov University), Moscow, Russia; ^9^Department of Pharmacy, College of Education, University of Anbar, AL Qaim, Iraq; ^10^Faculty of Pharmacy, Middle East University, Amman, Jordan; ^11^Department of Pharmacy, Al-Zahrawi University College, Karbala, Iraq; ^12^Venom and Biotherapeutics Molecules Lab, Medical Biotechnology Department, Biotechnology Research Center, Pasteur Institute of Iran, Tehran, Iran; ^13^Gastrointestinal and Liver Diseases Research Center, Iran University of Medical Sciences, Tehran, Iran

**Keywords:** ulcerative colitis, signaling pathways, probiotics, inflammation, gut microbiota

## Abstract

Ulcerative colitis (UC) is a chronic inflammatory bowel disease (IBD) marked by persistent inflammation of the mucosal lining of the large intestine, leading to debilitating symptoms and reduced quality of life. Emerging evidence suggests that an imbalance of the gut microbiota plays a crucial role in UC pathogenesis, and various signaling pathways are implicated in the dysregulated immune response. Probiotics are live microorganisms that confer health benefits to the host, have attracted significant attention for their potential to restore gut microbial balance and ameliorate inflammation in UC. Recent studies have elucidated the mechanisms by which probiotics modulate these signaling pathways, often by producing anti-inflammatory molecules and promoting regulatory immune cell function. For example, probiotics can inhibit the nuclear factor-κB (NF-κB) pathway by stabilizing Inhibitor of kappa B alpha (IκBα), dampening the production of proinflammatory cytokines. Similarly, probiotics can modulate the Janus kinase/signal transducer and activator of transcription (JAK/STAT) signaling pathway, suppressing the activation of STAT1 and STAT3 and thus reducing the inflammatory response. A better understanding of the underlying mechanisms of probiotics in modulating pathogenic signaling pathways in UC will pave the way for developing more effective probiotic-based therapies. In this review, we explore the mechanistic role of probiotics in the attenuation of pathogenic signaling pathways, including NF-κB, JAK/STAT, mitogen-activated protein kinases (MAPKs), Wnt/β-catenin, the nucleotide-binding domain (NOD)-, leucine-rich repeat (LRR)- and pyrin domain-containing protein 3 (NLRP3) inflammasome, Toll-like receptors (TLRs), interleukin-23 (IL-23)/IL-17 signaling pathway in UC.

## 1 Introduction

The prevalence of inflammatory bowel disease (IBD) is increasing in newly developed countries whose cultures have recently become increasingly Westernized. IBD is becoming more common around the globe; now, it affects 0.2% of the population in Europe (1, 2). IBD has two main subtypes: Crohn’s disease (CD) and ulcerative colitis (UC). CD is a long-term inflammatory condition of the intestines with an unknown cause, believed to arise from the interaction between environmental factors and genetic susceptibility in specific individuals (3, 4). CD is marked by ongoing intestinal damage and resulting disabilities. It can impact people of any age, from young children to older adults, causing considerable health issues and a reduced quality of life (5).

UC is a condition of unknown origin that leads to inflammation of the mucosa and submucosa in the colon and rectum, resulting in ulcer formation. A distinct boundary usually exists between healthy and affected intestinal tissue (6). Persistent surface mucous membrane inflammatory responses that extend from the rectum to the more distal portion of the colon are one of the histopathological indicators of UC. In UC, the mucosa is the only location of inflammatory lesions, and the muscular layer remains unaffected by injury (7). The pathophysiology of UC is complicated, including microbial dysbiosis, genetic, immunological, and environmental factors (8). Although medical treatment has advanced, managing UC is still tricky, necessitating other strategies. Immunosuppressants, corticosteroids, biological compounds, aminosalicylates, diets, and surgery are possible treatments, but they can only address the symptoms (9, 10). The incomplete understanding of the disease’s origins is undoubtedly to blame for the absence of effective therapy. Nevertheless, it is understood that UC has a multifaceted foundation based on a deficient immune reaction to luminal antigens and a faulty gastrointestinal barrier, which is altered by environmental factors in those with a hereditary predisposition (11–13).

Even though the origin of UC is unclear, evidence indicates that the onset and progression of the condition may be significantly influenced by gut microbial dysbiosis (14–16). It is thought that UC results from an imbalance between the mucosal immune system and the gut microbiota, leading to significant gut inflammation (17). It has been shown that in UC, there is an imbalance between the number of harmful and beneficial bacteria, which may cause the intestinal barrier to weaken and contribute to persistent inflammation (18). The immune system’s inappropriate response to dysbiotic alterations may exacerbate inflammatory processes and tissue damage in the colon (16, 19). Understanding how gut microbial dysbiosis contributes to UC might assist in developing novel treatment strategies that attempt to balance the gut microbiome and treat the symptoms of this crippling condition. Probiotics are being utilized more often to treat UC. Live nonpathogenic bacteria, including Lactobacillus, Bifidobacterium, and Enterococcus, are considered probiotics (20–25). Enhancing the function of the intestinal barrier, boosting local and systemic immunity, as well as restoring mucosal barrier integrity following the disruption are all possible due to the use of probiotics (7, 26). In addition, recent studies suggest that probiotics may reduce the severity of UC by modulating signaling pathways involved in the pathogenesis of the disease (27–31). In this study, we specifically focus on UC among the IBD for several reasons. UC presents a unique and well-defined set of pathophysiological features that are distinct from other forms of IBD, such as CD. Additionally, chronic inflammation and mucosal involvement in UC provide a specific context in which the modulation of probiotic signaling pathways can be studied in a detailed manner. Recent studies have highlighted the rising prevalence of UC, emphasizing the need for novel therapeutic approaches. Furthermore, with its specific challenges, the clinical management of UC benefits significantly from targeted research into the modulation of disease pathways by probiotics. This review aims to explore the potential of probiotics in mitigating significant pathogenic signaling pathways such as nuclear factor-κB (NF-κB), mitogen-activated protein kinase (MAPK), Janus kinase/signal transducer and activator of transcription (JAK–STAT), PI3K-Akt, Toll-like receptors (TLRs), interleukin-23 (IL-23)/IL-17, nucleotide-binding domain (NOD)-, leucine-rich repeat (LRR)- and pyrin domain-containing protein 3 (NLRP3), transforming growth factor beta (TGF-β) and Wnt/β-catenin pathway in UC, and to provide insights into the mechanisms by which these beneficial microorganisms can modulate the gut microbiome and promote intestinal barrier integrity.

## 2 The role of microbiome dysbiosis and probiotics in modulating signaling pathways in UC

Microbe-host communication is critical for maintaining vital host activities, and its disturbance has been linked to various disorders, including UC (15, 16). *Firmicutes*, *Bacteroidetes*, *Proteobacteria*, and *Actinobacteria* compose a large proportion of the gut bacterial phyla in healthy individuals, with estimates, generally, approximately 97% and significant inter-individual variations. Also, 90% of the microbiome’s entire makeup comprises *Firmicutes* and *Bacteroidetes*. These phyla synthesize short-chain fatty acids (SCFAs), particularly butyrate and propionate, from the fermentation of food elements such as indigestible fibers, and they are crucial for maintaining gut homeostasis. SCFAs have been demonstrated to be significant regulators of immunological homeostasis and energy sources for colonic mucosa cells (32). Several pieces of data support the importance of dysbiosis and the microbiome in the progression of UC. For instance, mice used in germ-free experiments mitigated colitis (33). In research utilizing mouse models, the transmission of bacterial strains linked to IBD results in gut inflammation in animals with certain genetic predispositions (34). Similarly, fecal transplantation from human donors with IBD to germ-free mice induces inflammation-promoting responses, with higher Th17 cell infiltration and pro-inflammatory mediators than transplants from healthy human donors (35). Additionally, many investigations have been devoted to defining the dysbiotic state observed in the gut microbiome of UC patients, declaring a decline in bacterial diversity along with a decrease in relative amounts of *Enterococcus* and *Bacteroides*, and in members of *Clostridium* subcluster XIVa, as well as increases in several opportunistic pathogenic bacteria (36). There is evidence that several IBD phenotypes may be impacted by changes in microbiome composition (37). For instance, *Ruminococcus gnavus* group and *Fusobacterium nucleatum* were shown to be substantially more prevalent in CD when compared to controls, according to research by Clooney and colleagues. On the other hand, CD had lower levels of *Ruminococcus albus*, *Eubacterium rectale*group, and *Faecalibacterium prausnitzii*. Compared to controls, *Eubacterium* and *Roseburia* were two of the most significant genera in categorizing CD or UC (37, 38). In other words, dysbiosis may decrease crucial processes required for preserving the integrity of the intestinal barrier and gut equilibrium. Hence, a dysbiotic microenvironment may be to blame for changes in the immune response and inflammation-promoting activity.

**Figure 1:**
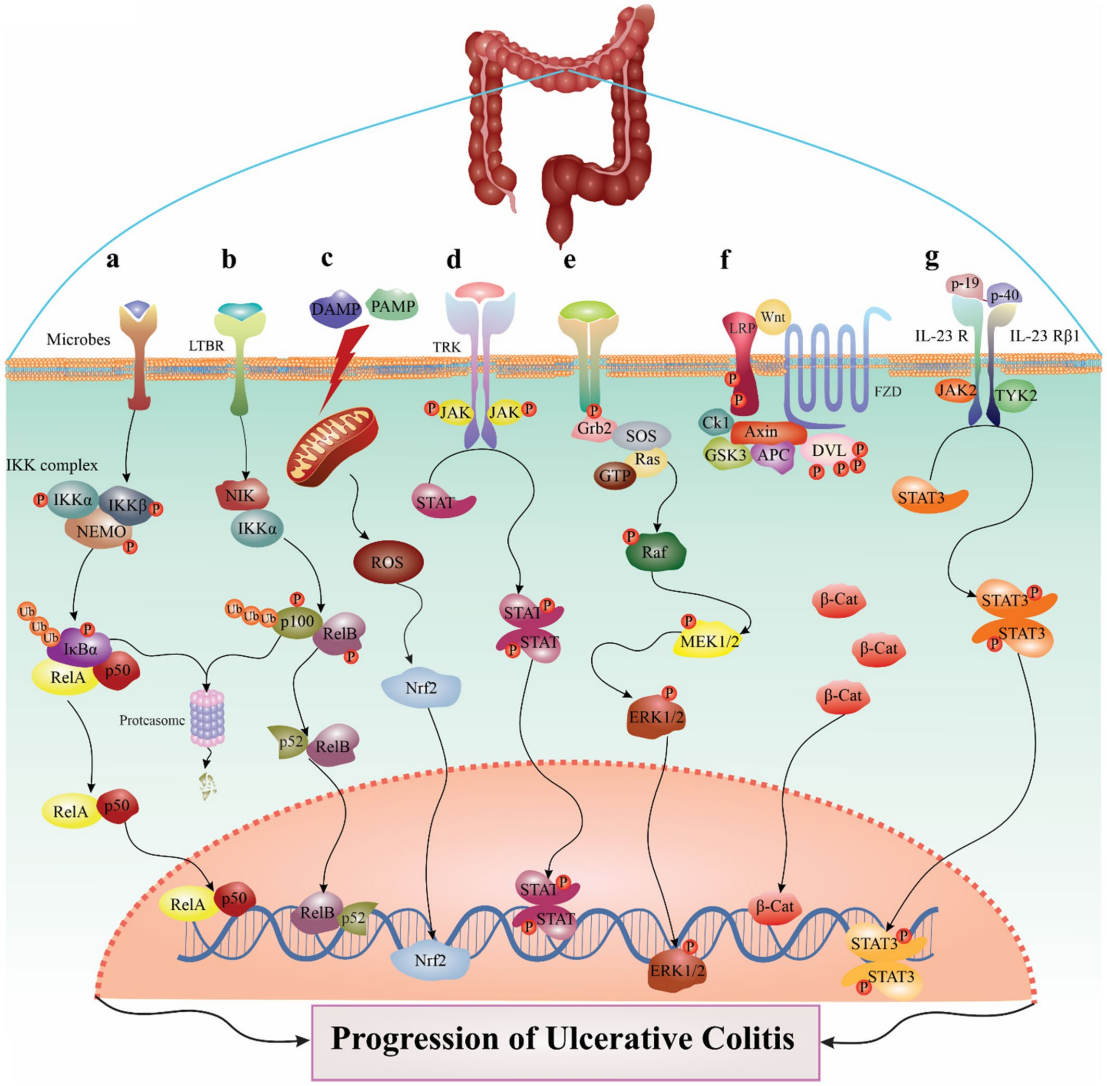
The major signaling pathways involved in the pathogenesis of ulcerative colitis. For example, in NF-κB pathway, several stimuli could trigger the canonical route **(A)**, and the IKK complex, which is comprised of IKKα, IKKβ, and NEMO, phosphorylates IκBα inducible to cause its destruction. IKK may be triggered by various events, including microbes, stress agents, mitogens, cytokines, and growth factors. When IKK is activated, it phosphorylates IκBα, which causes the nuclear translocation of canonical NF-κB members—most notably the p50/RelA and p50/c-Rel dimers. Also, LTβR, BAFFR, CD40, and RANK ligands are among the stimuli that might trigger the noncanonical NF-κB pathway **(B)**. Noncanonical NF-B induction, in contrast to the canonical route, depends on processing the NF-κB2 precursor protein, p100. An essential signaling protein for the noncanonical route, NF-κB-inducing kinase (NIK) collaborates with IKKα to induce p100 ubiquitination, phosphorylation, and processing. This causes the nuclear translocation of the noncanonical NF-κB complex p52/RelB and the production of mature NF-κB2 p52. The noncanonical route collaborates with the canonical pathway to control certain adaptive immunity activities. Another signaling pathway described in detail in the manuscript. **(C)** NLRP3 pathway, **(D)** JAK/STAT pathway, **(E)** MAPK pathway, **(F)** Wnt/β-catenin pathway, **(G)** IL-23/IL-17 signaling pathway.

Probiotics are living bacteria that, when taken in sufficient quantities, may positively affect health (39–42). Intestinal microbial community modification, pathogen restriction, immune system regulation, promotion of epithelial cell proliferation and differentiation, and reinforcement of the intestinal barrier are some of the mechanisms of probiotics (Figure 1) (43). Modifications in the structure and functioning of the gut microbiome are some of the probiotic’s hypothesized mechanisms. Probiotics create metabolic chemicals or antimicrobial substances that inhibit the development of other microorganisms (44, 45) or could compete with other intestinal microorganisms for receptors and binding sites on the gastrointestinal mucosa (46). Probiotic Lactobacillus strains have been shown to improve intestinal barrier function, which may prevent gastrointestinal infections, IBS, and IBD (46).

Additionally, probiotics may modify intestinal immunity and change how immune cells and intestinal epithelia react to bacteria in the gut lumen (43, 47). Probiotics’ impacts on the diversity, arrangement, and performance of the gut microbiota have been researched using a variety of methodologies, including targeted, culture-dependent approaches and metagenomic sequencing. Nevertheless, a few studies have shown relationships between probiotic administration and modified microbiome (48). Several different probiotics have been investigated for their ability to modify gut flora and alleviate symptoms of UC. For instance, research on the probiotic *Lactiplantibacillus plantarum* (*L. plantarum*) has demonstrated that it helps UC patients’ barrier activity and reduces the inflammatory response (49). Another probiotic cocktail that has displayed promise in reducing inflammatory processes and improving UC symptoms is VSL#3. This cocktail consists of eight distinct bacterial strains (50).

Recently, research studies have also been conducted on the role of fungal probiotics in gastrointestinal diseases such as IBD (51–55). In a recent study, Gravina et al. explored the anti-inflammatory properties of the nutraceutical compound HBQ-Complex^®^—comprising *Hericium erinaceus*, berberine, and quercetin—combined with the vitamins biotin (B7) and niacin (B3) in patients with IBD (53). *H. erinaceus* classified under Agaricomycetes in the phylum Basidiomycota, is a Chinese medicinal and edible fungus predominantly found in East Asia. It has a long history of use in traditional Chinese medicine (TCM), spanning several centuries (56). The results indicate that a combination of *H. erinaceus*, berberine, quercetin, biotin, and niacin exhibits strong anti-inflammatory effects on inflamed IBD tissues. The HBQ-Complex®, when paired with biotin and niacin, significantly reduces the expression of key proinflammatory cytokines (cyclooxygenase-2 [COX-2] and tumor necrosis factor alpha [TNF-α]) at both the mRNA and protein levels in *ex vivo* IBD tissue (53). This suggests that the compound can mitigate the inflammatory burden, potentially leading to decreased inflammation and tissue damage. An increase in IL-10 expression suggests a more effective anti-inflammatory response. IL-10 is well-known for regulating immune responses and maintaining intestinal homeostasis. The progressive rise in IL-10 levels indicates that the HBQ-Complex^®^ not only reduces inflammation but also creates an anti-inflammatory environment that promotes tissue repair and regeneration (53). While the study’s *ex vivo* results suggest that the nutraceutical ingredient may effectively reduce inflammation in IBD, it is essential to emphasize that these findings need to be confirmed *in vivo*. Clinical trials are necessary to evaluate this compound’s efficacy, dosage, and safety in human IBD patients.

Diling et al. investigated the efficacy of *H. erinaceus* extracts in treating IBD using a rat model. Various *H. erinaceus* extracts—including polysaccharide, alcoholic, and whole extracts—were administered to rats with IBD induced by 2,4,6-trinitrobenzene sulfonic acid (TNBS) (51). The results suggest that *H. erinaceus* extracts exert a multifaceted therapeutic effect on IBD, primarily by modulating the immune system and gut microbiota. These extracts enhance the levels of anti-inflammatory cytokines (such as IL-10 and Foxp3) while reducing proinflammatory markers (such as NF-κB p65 and TNF-α), indicating a robust anti-inflammatory response (51). Increased T cell activation was identified, notably in the group treated with alcoholic extracts (51). Increased T cell activation, particularly with the alcoholic extracts, suggests a significant immune-modulatory effect that could enhance the body’s ability to manage inflammation. Additionally, Diling et al. found that the polysaccharides in *H. erinaceus* extracts promote the growth of beneficial gut bacteria, which may aid in restoring a healthy balance of gut microbiota disrupted by IBD (51). Alcoholic extracts possess direct antimicrobial properties and modulate immune responses, which may help reduce pathogenic bacterial load and enhance overall gut health (51). The research indicates that *H. erinaceus* extracts may significantly improve both clinical and histological outcomes in IBD, highlighting their potential as a supplementary treatment for managing the disease in individuals (51).

**Figure 2:**
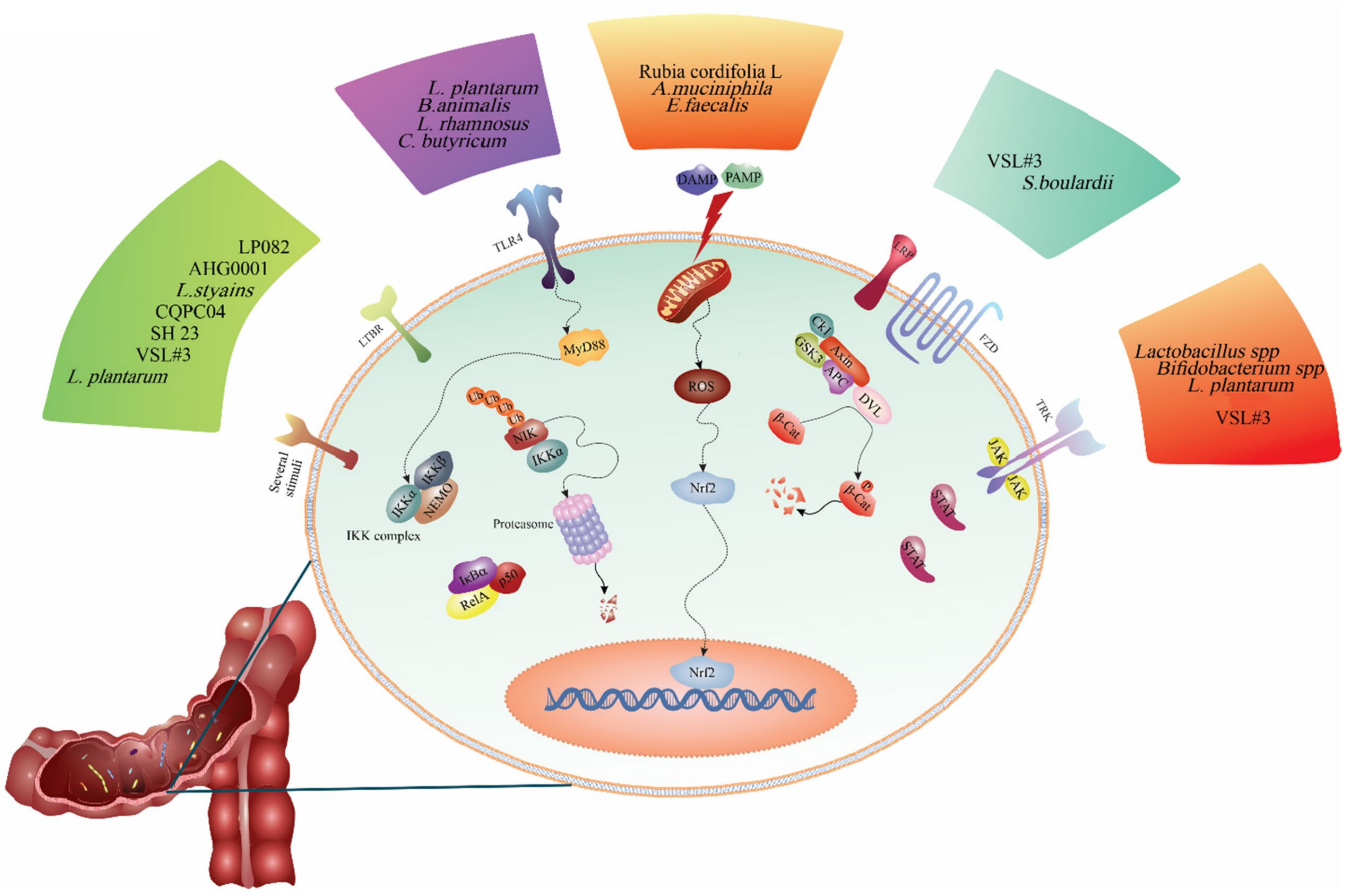
The regulatory role of probiotics on the signaling pathways involved in the pathogenesis of ulcerative colitis. As shown in the figure, different probiotic strains modulate various signaling pathways. For example, probiotics such as *Lactobacillus casei* ATCC 393, *Akkermansia muciniphila*, *Enterococcus faecalis*, and *Rubia cordifolia* L. alleviates ulcerative colitis are inhibiting the NLRP3 signaling pathway. Other probiotics such as *L. plantarum* AR113 and VSL#3, *L. plantarum* L15, and *Bifidobacterium animalis* subsp. Lactis mitigates ulcerative colitis by reducing inflammation in the colon and decreasing TLR4/MyD88/NF-κB pathway expression.

Probiotics have been shown to exert beneficial effects on the host in UC primarily through the modulation of various signaling pathways, such as NF-κB, MAPK, TLR, JAK/STAT, Wnt/β-catenin, and TGF-β, in addition to their impact on the gut microbiome (57–60). Recently, mechanistic investigations showed that probiotics modulate various signaling pathways involved in the pathogenesis of UC, such as NF-κB (30, 60, 61), MAPK (62), JAK–STAT (63), PI3K-Akt (64), Wnt/β-Catenin (65), TLR signaling pathway (66), NLRP3 inflammasome (29), and also IL-23/IL-17 axis (67, 68). For instance, the NF-κB transcription factor is essential for inflammatory responses and the immune system. Probiotics have been shown to reduce UC inflammatory response reactions by modulating the NF-κB signaling pathway (31, 61, 69). Next, we will examine the role of the signaling pathways involved in the pathogenesis of UC and discuss the findings related to the role of probiotics in modulating these signaling pathways (Figure 2).

## 3 The overview of inflammatory signaling pathways involved in UC

### 3.1 The role of NF-κB pathway in UC

The NF-κB pathway is an essential signaling mechanism that controls inflammatory and immunological reactions (70, 71). Despite having different signaling mechanisms, the canonical and noncanonical pathways, both implicated in the activation of NF-κB, are crucial for controlling immunological and inflammatory processes; for more details, refer to Sun et al. (72) and Vallabhapurapu et al. (73). The canonical and noncanonical pathways of NF-κB are the two main signaling mechanisms that control immunological and inflammatory processes. Several stimuli could trigger the canonical route (74), and the IκB kinase (IKK) complex, which is comprised of IKKα, IKKβ, and non-catalytic accessory scaffold protein IKKγ (non-catalytic accessory scaffold protein IKKγ [NEMO]), phosphorylates IκBα inducible to cause its destruction (75). IKK may be triggered by various events, including microbes, stress agents, mitogens, cytokines, and growth factors (76). When IKK is activated, it phosphorylates IκBα, which causes the nuclear translocation of canonical NF-κB members—most notably the p50/RelA and p50/c-Rel dimers (77, 78). Lymphotoxin beta receptor (LTβR), BAFFR, CD40, and RANK ligands are among the stimuli that might trigger the noncanonical NF-κB pathway (71, 72). Noncanonical NF-κB induction, in contrast to the canonical route, depends on processing the NF-κB2 precursor protein, p100. An essential signaling protein for the noncanonical route, NF-κB-inducing kinase (NIK) collaborates with IKKα to induce p100 ubiquitination, phosphorylation, and processing (79, 80). This causes the nuclear translocation of the noncanonical NF-κB complex p52/RelB and the production of mature NF-κB2 p52 (72, 81). The noncanonical route collaborates with the canonical pathway to control certain adaptive immunity activities (82).

Numerous studies indicate that the NF-κB pathway is crucial in controlling the production of cytokines in patients with UC and the inflammatory responses and immune system reactions in the gastrointestinal tract of UC, even if the mechanisms are still unclear currently (83–85). Patients with UC have a greater than normal expression of NF-κB p65 in their lamina propria monocytes, intestinal mucosal epithelium, and crypt epithelial cells. NF-κB is mainly expressed in the nucleus rather than the cytoplasm (86). In lamina propria mononuclear cells from patients with UC, NF-κB p65 antisense oligonucleotides inhibit the NF-κB pathway and suppress NF-κB-dependent IL-8 mRNA expression and IL-1β, reducing proinflammatory cytokine production (87). The genetic insufficiency of negative NF-κB pathway regulators, including the deubiquitinases CYLD and A20, which are known to induce colonic inflammatory processes, has also been shown in an array of additional animal model investigations (88, 89). According to these results, colitis brought on by TNBS and dextran sulfate sodium (DSS) is lessened by decoy oligonucleotides that target the DNA-binding function of NF-κB proteins (90, 91). Experimental colitis and colitis-associated malignancies are suppressed by deleting IKKβ in myeloid cells (92). These results align with the notion that NF-κB plays a role in mediating the production of cytokines that promote inflammation by innate immunity cells and the development of the Th1 and Th17 inflammatory T cell subsets. NF-κB has protective effects in gastrointestinal epithelial cells, which is necessary for preserving intestinal immunological homeostasis and epithelial integrity, compared with its proinflammatory effect in myeloid cells (93, 94). Gastrointestinal epithelial cells lacking NEMO, IKKβ, or both IKKβ and IKKα spontaneously generate long-lasting inflammation in mice (94, 95). Since NF-κB serves different purposes in innate immune cells and epithelial cells, it is inappropriate activation or genetic deficit might contribute to the etiology of UC.

### 3.2 The role of NLRP3 inflammasome pathway in UC

The development of several inflammatory diseases, such as UC, has been linked to the NLRP signaling pathway, which is a vital part of innate immunity (96). The innate immunity, which identifies and reacts to invasive infections and injured tissues, includes the NLRP signaling pathway. Danger-associated molecular patterns (DAMPs) and pathogen-associated molecular patterns (PAMPs) are two triggers that cause the NLRP pathway to become activated (97). An example of a pattern recognition receptor (PRR), the NLRP3 inflammasome, is a multiprotein complex that plays a crucial role in the host’s innate immunity against microbial, fungal, and viral infections (98–102). The inflammasome is a complex of multiple proteins that forms when the NLRP3 pathway is active. The inflammasome then causes caspase-1 to be activated and the production of cytokines that cause inflammation, notably IL-1β and IL-18 (103). The NLRP pathway undergoes disruption in UC, which results in increased inflammasome activation and the production of proinflammatory cytokines. Several studies have demonstrated that the colonic mucosa of UC patients has higher levels of NLRP3, a crucial element of the NLRP pathway. Genetic variations in the NLRP3 gene have also been linked to a higher risk of having UC (104–106). It has been shown that the NLRP3 inflammasome plays a significant role in the pathological hallmarks of UC. Potential prospects for treating the UC issue include using probiotics, chemical agents, phytochemicals, and plants to target NLRP3 inflammasome signals (96). In summary, the NLRP signaling pathway is dysregulated and thus significantly contributes to the pathophysiology of UC. The activation of NLRPs is a key feature of UC, and understanding the processes behind this activation might pave the way for developing new therapeutic approaches.

### 3.3 The role of JAK–STAT pathway in UC

JAK1, JAK2, JAK3, and tyrosine kinase 2 (TYK2), which transduce cytokine-mediated signals through the STAT pathway, are components of the JAK–STAT system. This key signaling mechanism contributes to the control of the immune system’s responses (107). Upon ligand-mediated receptor multimerization, JAK stimulation starts with two JAKs nearby, enabling trans-phosphorylation. Further targets, such as the receptors and STATs, are later phosphorylated by the activated JAKs (108). Dimerization of phosphorylated STATs precedes their translocation across the nuclear membrane and subsequent regulation of gene expression (109). Seven different members of the STAT family have distinct biological functions (110, 111). Both STAT1 and STAT2 are involved in interferon signaling (108, 111), T helper cell development requires STAT3, IL-12 signaling requires STAT4, Th 1 cell differentiation requires STAT6, and IL-4 and IL-13 signaling through STAT6 is implicated in allergic responses (112, 113). JAK1/JAK2 knockout mice experience perinatal death, demonstrating the significance of JAK/STAT members for cytokine signaling in knockout genetic experiments (114, 115), Mice lacking JAK3/TYK2/STAT6 have impaired immunity and are more prone to diseases (116, 117), and mice deficient in STAT5A/STAT5b are sterile and die at an early age from severe anemia (118).

JAK antagonists are a novel treatment for IBD. JAK antagonists, the first oral small molecule treatment for IBD, are used. They act rapidly and may cause an immediate clinical response due to rapid entry into the systemic circulation (119). JAK antagonists prevent JAK from being phosphorylated, hence inhibiting downstream JAK–STAT signaling. It is noteworthy that specific JAK receptors exhibit selectivity for various cytokines. IL-5, IL-9, IL-13, and IL-33 for UC, IL-10, IL-12, IL-27, and interferon (IFN)-γ for CD, and IL-6, IL-12, IL-17, IL-21, IL-23, and TNF-α for both IBD types are cytokines linked to disease etiology (120). Several cytokines may activate the JAK–STAT pathway. For instance, IL-23 stimulates the STAT3 route via JAK2, while TYK2 promotes the STAT4 pathway (121). Additionally, JAK1, JAK2, and TYK2 are used by IL-6 to activate the STAT3 pathway (122). Targeting the JAK–STAT pathway offers a variety of treatment options since each JAK has a distinct impact. In clinical studies, targeting this system with JAK antagonists for the medical management of UC has demonstrated promise since it may decrease inflammatory responses and improve manifestations (121). Further studies are warranted to completely comprehend the significance of the JAK–STAT pathway in UC and the long-term impact of JAK antagonists on patients. Nevertheless, the usage of JAK antagonists might have adverse consequences.

### 3.4 The role of the MAPK pathway in UC

The MAPKs are a diverse collection of enzymes that phosphorylate the amino acids serine and threonine in several proteins. At present, seven distinct MAPK families have been identified: extracellular regulated kinase 1/2 (ERK1/2), extracellular regulated kinase 3/4 (ERK3/4), extracellular regulated kinase 5 (ERK5), extracellular regulated kinase 7/8 (ERK7/8), p38 kinase, Nemo-like kinase (NLK) and the c-Jun N-terminal kinase (JNK) group (123). The signaling pathways that the members of these families affect may not overlap or be independent of one another. Growth factors, oxidative stress, cytokines that promote inflammatory processes, and other stimuli may all activate the MAPK pathway. When MAPKs are activated, they move to the nucleus and control gene expression, influencing cellular responses such as apoptosis, cell division, and proliferation (124). A traditional mechanism for MAPK signaling is the ERK pathway (125). JNK and p38 kinase are two other MAPK family members in addition to ERK1/2 that have been implicated in inducing apoptosis (126). It has been demonstrated that the inflamed colonic mucosa of UC patients exhibits upregulation of many MAPKs, including ERK, JNK, and p38 MAPK. TNF-α, IL-1β, and IL-6 are cytokines that are increased in UC patients’ inflamed mucosa and trigger these MAPKs (127). To attract and activate immune cells and maintain an inflammatory process, activated MAPKs have been discovered found to boost the generation of cytokines that are proinflammatory, chemokines, and adhesion molecules (128, 129). Modulation of MAPKs has been shown in preclinical research to decrease inflammation and improve colitis in animal models (62). It is challenging to anticipate the consequences of MAPK suppression since MAPKs may activate various downstream signaling pathways, and their function can be modulated through various feedback mechanisms. Additionally, the MAPK pathway plays a role in various physiological activities, such as cell division and proliferation (124); inhibiting it might have unfavorable side effects. Despite these obstacles, the MAPK pathway remains a promising therapeutic target for UC. Combination medications targeting various pathways, including the JAK–STAT and MAPK pathways, might be effective in helping UC patients achieve their ideal results. Further studies are required to determine the most appropriate targets to discover safe and effective treatments for this crippling condition.

### 3.5 The role of the Wnt/β-catenin pathway in UC

A critical signaling mechanism in controlling cell growth and differentiation is the Wnt/β-catenin pathway. This pathway’s improper regulation has been linked to the etiology of many disorders, including UC (130). Given the significant role Wnt signaling plays in repairing tissues and cell proliferation, it is reasonable to predict that colitis will cause significant worldwide alterations in Wnt molecules that promote mucosal renewal. Nevertheless, there is not much data to support this. In contrast to healthy controls, You et al. discovered alterations in the expression of many Wnt ligands and receptors in UC samples (131); however, the alterations were unrelated to disease activity or Wnt target gene expression, and further research could not confirm these results (132). Nevertheless, chronic IBD and animal models exhibit Wnt/β-catenin pathway activation (133). Signaling via epithelial TNF receptors encourages mucosal healing in patients with IBD (134). In the intestinal tract, Wnt ligand expression is extremely segregated (135). This issue of differentiation and prior evidence provide a comprehensive analysis of non-epithelial Wnt origins in the gut and their function in tissue equilibrium (136, 137). However, advances in this area suggest that throughout colitis, inflammatory mediators are responsible for epithelial maintenance and renewal. For UC patients, targeting Wnt/β-catenin may have the potential to achieve the most favorable outcomes. Ultimately, a better understanding of the role of the Wnt/β-catenin pathway in UC will enable the development of more targeted and effective therapies to improve patient outcomes.

### 3.6 The role of IL-23/IL-17 pathway in UC

According to reports, the pathogenesis of UC is significantly influenced by the IL-23/IL-17 axis, which supports Th17 cells and cytokine-related immune response. Blocking IL-23/IL-17 pathways is receiving much attention as a potential therapy for some chronic inflammatory conditions (138). Increased IL-23 production has been shown in many mouse models of colitis (139–141). Additionally, elevated levels of IL-17 have been observed in various animal models of colitis (142–144). Therefore, despite the variety of the sample size and disease severity, all observational investigations revealed elevated levels of IL-23 and IL-17A in the blood of patients with UC (145–150). Increased IL-23 levels were associated with a more severe disease course in UC subjects (145). However, results on the expression of IL-23 and IL-17 in the inflamed mucosa are still debatable, perhaps due to the heterogeneity in sample size, clinical and endoscopic activity, and biopsy region (146, 147, 151). Additionally, in many mice models of colitis, the inhibition of IL-23 and, to a lesser degree, IL-17 significantly reduced intestinal inflammation (141, 142, 152, 153). These findings provided more evidence for the role of IL-23 and IL-17 in UC pathogenesis, prompting the proposal of techniques to suppress these cytokines as a potential therapy for UC. Overall, the IL-23/IL-17 signaling pathway plays a vital role in the pathogenesis of UC, and targeting this pathway may represent a promising therapeutic strategy for treating this disease. However, further research is needed to fully understand the complex interactions between this pathway and other signaling pathways involved in UC and to develop safe and effective therapies that target this pathway in patients with UC.

## 4 Mechanisms of probiotic-mediated signaling pathway modulation in UC

### 4.1 The role of probiotics in the modulation of the NF-κB signaling in UC

Hegazi et al. discovered colonic mucosal damage and inflammation in patients with UC using hematoxylin and eosin staining, as well as an upsurge in colonic myeloperoxidase (MPO) activity, fecal calprotectin, and expression of colonic TNF-α and NF-κB p65 proteins. They found that after a period of 8 weeks of probiotics (*Lactobacillus delbruekii* and *Lactobacillus fermentum* [*L. fermentum*]), there was a significant reduction in inflammatory responses, as evidenced by a reduction in colonic IL-6 level, TNF-α and NF-κB p65 expression, recruitment of leukocytes (as evidenced by decreased colonic MPO activity), and fecal calprotectin levels when contrasted with both the sulfosalt (83). These findings aligned with the discovery that *Bifidobacterium longum* suppresses NF-κB activation and decreases TNF-α and IL-8 generation in the inflamed mucosa of active UC without affecting colonic cell survival (154). Zhou and colleagues assessed the *in-vitro* impact of *Limosilactobacillus fermentum* (CQPC04) on DSS-induced colitis mice (31). This research discovered an intriguing phenomenon: *L. fermentum* CQPC04 could suppress the activation of NF-κBp65 in colitis mouse colon tissue, and the inhibitory effect improved with increasing *L. fermentum* CQPC04 level. NF-κB is a transcription factor that plays a vital role in the immune response process. In an inactive state, it interacts with its inhibitory protein, IκB (31). Furthermore, they discovered that DSS modeling enhanced the phosphorylation of the IκB protein, which triggers NF-κB (31). Earlier studies have shown that TNF-α may activate NF-κB in various cell types (155). They discovered that the concentrations of TNF-α and interferon gamma (IFN-γ) in the blood of colitic animals were significantly raised, as were their mRNA and protein expression in colon tissues. These indications dropped dramatically in blood, mRNA expression, and protein expression in colon tissues when colitis animals were treated with *L. fermentum* CQPC04 by oral gavage. These indications were significantly lower in the LF + H + DSS group, demonstrating that *L. fermentum* CQPC04 controls TNF-α and IFN-γ expression in colitis animals, minimizing damage caused by inflammation (31). Furthermore, it was previously established that IL-1, IL-6, IL-12, COX-2, and Inducible nitric oxide synthase (iNOS) are cytokines that promote inflammation implicated in NF-κB activation (156). *L. fermentum* CQPC04 inhibited the expression of IL-1, IL-6, IL-12, COX-2, and iNOS in colitis mice at the serum, messenger RNA (mRNA), and protein levels, lowering the inflammation-causing injury of mediators of inflammation and exhibiting a prophylactic impact on the onset of colitis, according to the findings of Zhou et al. (31). Furthermore, IL-10 has been shown to lower MPO activity, block NF-κB activation in inflammatory cells, increase the IL-1RA/IL-1 ratio, and suppress other cytokines that promote inflammation (157). Eventually, Zhou and collaborators discovered that IL-10 concentrations in the blood and tissues of mice with DSS-induced colitis declined to various degrees but rose in serum and colon tissues following *L. fermentum* CQPC04 treatment (31). According to this research, the method by which *L. fermentum* CQPC04 improves the symptoms of DSS-induced colitis in mice might be connected to the NF-κB signaling system.

In another investigation, the researcher’s Wu et al. investigated the therapeutic efficacy and mechanism of the probiotic *L. plantarum HNU082* (*Lp082*) on UC (60). The typical intestinal mucosal barrier consists of mechanical, chemical, immunological, and biological barriers. *Lp082* demonstrates high effectiveness in treating UC, which drives us to investigate its mechanism of action deeper. According to the findings of this research, Lp082 may strengthen the mucosal barrier of the intestinal tract by enhancing the biological, chemical, mechanical, and immunological barriers synergistically, consequently reducing UC. Lp082’s possible strategies for treating UC include improving the intestinal mucosal barrier, modulating inflammatory processes, and affecting neutrophil recruitment (60). According to the findings of Wu et al., *Lp082* may control inflammatory variables to preserve the balance between regulatory T cells and effector T cells to modulate gastrointestinal mucosal immunity, therefore preserving the intestinal mucosal barrier in conjunction with reducing inflammatory responses by suppressing the NF-κB pathway (60). Their results demonstrated that Lp082 enhanced the immunological barrier by reducing the levels of proinflammatory cytokines (IL-1, IL-6, TNF-α, MPO, and IFN-γ) while increasing the levels of anti-inflammatory cytokines (IL-10, TGF-β1, and TGF-β2) and suppressing the NF-κB signaling pathway. In summary, *Lp082* may help maintain the intestinal mucosal barrier, reduce inflammation, and modulate microbial imbalances in a mouse model of DSS-induced colitis.

Zong and colleagues used a mouse model of colitis produced by DSS to assess the anti-inflammatory activity of LPxTG-motif surface protein (LMP) generated from *Limosilactobacillus reuteri SH 23* (158). The findings demonstrated that LMP inhibits DSS-induced ulcerative colitis in mice through the MAPK-dependent NF-κB pathway. In the LMP-treated DSS mice model, the inflammatory factors such as IL-6 and TNF-α were reduced, but IL-10 production was increased. A beneficial relationship exists between the IL-10 cytokines and modifications in the intestinal microbiota *Lactobacillus* and *Akkermansia* genus in the LMP-treated mice groups. This treatment also affected the diversity of the bacteria in the intestinal microbiota in this group, such as an upsurge in the abundance of these genera (158). As a result, LMP produced from *L. reuteri SH 23* can potentially ameliorate inflammation-related diseases by balancing intestinal flora and inhibiting inflammatory mediators in the NF-κB pathway (Table 1).

**Table 1:** The regulatory role of various probiotic strains in modulating signaling pathways involved in the pathogenesis of ulcerative colitis.

UC	Study type	Probiotic strains	Molecular targets	Mechanisms and conclusion	References
	DSS-induced UC	*Lactobacillus plantarum HNU082* (*Lp082*)	NF-κB signaling pathway	Lp082 protects mice against DSS-induced colitis by lowering IL-1β, IL-6, TNF-α, MPO, and IFN-γ levels while boosting IL-10, TGF-β1, and TGF-β2 and blocking the NF-κB signaling pathway	Wu et al. (60)
	HT-29 and Caco2	*Lactobacillus* strains	NF-κB signaling pathway	*Lactobacillus strains* inhibit Salmonella-induced inflammatory responses by modulating TLR-negative regulators and the NF-κB pathway	Kanmani and Kim (69)
	DSS-induced UC	*Lactobacillus fermentum CQPC04*	NF-κB signaling pathway	*L. fermentum* CQPC04 dramatically decreased the expression of NF-κBp65, TNF-α, IL-1β, COX-2, and iNOS in mice colon tissues while increasing the expression of IκB- and SOD2	Zhou et al. (31)
	Human sample (colonic tissue and stool)	*Lactobacillus delbruekii* and *L. fermentum*	NF-κB signaling pathway	The NF-κB signaling cascade and inflammatory processes may be suppressed by oral probiotic supplementation, which may aid in sustaining remission and avoiding the recurrence of UC	Hegazy and El-Bedewy (83)
	DSS-induced UC	*Limosilactobacillus reuteri SH 23*	NF-κB pathway	The LPxTG-motif surface protein (LMP) isolated from *L. reuteri* SH 23 has been shown to reduce inflammation by restoring intestinal flora balance and blocking NF-κB-mediated inflammatory responses	Zong et al. (158)
	HT-29	*Escherichia coli Nissle1917*	TLR1, 3, 7 decreased, TLR2 increased	An *E. coli Nissle1917*-derived metabolite modulates key mediator components in the TLR signaling pathway	Damoogh et al. (66)
	Rat model of colitis	*Lactobacillus acidophilus*	TLR9	Integrating probiotics with traditional Chinese medicine (TCM) is an innovative approach that has shown promise in treating UC because it modulates the gut microbiota and its metabolites, TLR9, and cytokines via many mechanisms	Aximujiang et al. (183)
	DSS-induced UC	*Lactobacillus casei*	TLR-4	In the absence of TLR-4 complex signaling, *L. casei* modifies the production of inflammatory mediators and reduces neutrophilic recruitment	Chung et al. (163)
	DSS-induced UC	*L. plantarum* AR113 and *L. casei* AR342	TLR4/MyD88/NF-κB pathway	Probiotics reduce inflammation in the colon of mice by increasing HO-1 expression and decreasing TLR4/MyD88/NF-κB pathway expression	Xia et al. (177)
	Rat model of colitis	VSL#3	TLR4-NF-κB signal pathway	VSL#3 reduces the production of NF-κB and TNF-α in rats with colitis through the TLR4-NF-κB signal pathway, making it a first-choice medication for colitis therapy	Hui et al. (187)
	DSS-induced UC	*L. plantarum* L15	TLR4/MyD88/NF-κB pathway	Since the actions of *L. plantarum L15* decreased the expression of TLR4 and MyD88 genes and genes linked with the NF-κB signaling pathway, this strain shows promise as a novel probiotic with potential use for treating UC	Yu et al. (30)
	DSS-induced UC	*Bifidobacterium animalis* subsp. *lactis*	TLR4/MyD88/NF-κB pathway	*B. lactis XLTG11* might mitigate DSS-induced colitis by suppressing the TLR4/MYD88/NF-κB signaling pathway induction, controlling inflammatory mediators, enhancing intestinal barrier integrity, and altering the gut microbiota	Li et al. (68)
	DSS-induced UC	*Lactobacillus rhamnosus GG HM0539*	TLR4/MyD88/NF-кB Axis	HM0539 significantly reduced the generation of inflammatory compounds linked to the TLR4/Myd88/NF-κB axis activation in colon tissue	Wang et al. (182)
	DSS-induced UC	*Lactobacillus casei ATCC 393*	NLRP3-(Caspase-1)/IL-1β signaling pathway	*L. casei ATCC 393* and its metabolites reduced the NLRP3-(Caspase-1)/IL-1β signaling pathway-mediated ulcerative inflammatory reaction in C57BL/6 mice that DSS had provoked	Dou et al. (195)
	DSS-induced UC	*Enterococcus faecalis*	NLRP3	*E. faecalis* might be an effective and safe treatment for reducing NLRP3-mediated colitis and inflammation-related colon tumorigenesis	Chung et al. (194)
	DSS-induced UC	*VSL#3*	Wnt/β-catenin pathway	VSL#3 probiotic combination inhibits UC-associated tumorigenesis in mice and cells by modulating the inflammatory and Wnt/β-catenin pathways	Li et al. (222)
	Rat model of colitis	*Saccharomyces boulardii*	Wnt/β-catenin signaling	In combination with sulfasalazine (salicylazosulfapyridine [SASP]), probiotics reduce inflammatory responses by blocking the triggering of the Wnt/β-catenin signaling pathway, enhancing intestinal function and repairing intestinal integrity	Dong et al. (65)
	HT-29 cell line	*Lactobacillus* spp. and *Bifidobacterium* spp.	NF-κB and JAK/STAT signaling pathways	*Lactobacillus* spp. and *Bifidobacterium* spp. inhibited HT-29 cell inflammation by regulating the JAK/STAT and NF-κB pathways	Aghamohammad et al. (27)
	DSS-induced UC and HT-29 cell	*Lactobacillus plantarum-12*	JAK–STAT	*L. plantarum-12* may reduce DSS-induced UC by reducing intestinal inflammation and reestablishing the altered gut flora. *L. plantarum-12* may be helpful as a probiotic to treat colitis	Sun et al. (63)
	DSS-induced UC	*VSL#3*	STAT3 pathway	Downregulation of IL-6, IL-23, STAT3, and P-STAT3 expression in colonic tissue by live and heat-killed VSL#3. Heat-killed VSL#3 inhibits the STAT3 pathway and exerts significant beneficial anti-inflammatory properties	Sang et al. (249)
	DSS-induced UC	*L. plantarum* subsp. *plantarum SC-5*	NF-κB and MAPK signaling pathways	*L. plantarum* supsp. *plantarum SC-5* reduced inflammation by reducing the protein production of the NF-κB and MAPK signaling pathways	Shi et al. (160)
	DSS-induced UC	*L. fermentum ZS40*	NF-κB and MAPK pathways	*L. fermentum ZS40* decreased the relative expression of p38 and JNK1/2 mRNA as well as p38, p-p38, JNK1/2, and p-JNK1/2 proteins through the NF-κB and MAPK pathways	Chen et al. (62)
	DSS-induced UC	*L. acidophilus*	IL-23/Th17 axis	*L. acidophilus* inhibited Th17 cell-mediated production of the proinflammatory mediator IL-17 by downregulating IL-23 and TGF-β1 expression and phosphorylating p-STAT3	Chen et al. (57)
		*Lactobacillus and Bifidobacterium Strains*	IL-23/Th17 axis	Probiotic *Lactobacillus* and *Bifidobacterium* strains reduce AIEC virulence and inhibit the IL-23/Th17 axis in UC but not Crohn’s disease	Leccese et al. (67)
	TNBS	*Faecalibacterium prausnitzii*	IL-17	*F. prausnitzii* reduces rat colorectal colitis by blocking interleukin-17	Zhang et al. (250)
	TNBS	*L. acidophilus*	IL-17	Oral administration of *L. acidophilus* stimulates an IL-17-dependent innate protection reaction, activates innate lymphoid cells type 3, and alleviates colitis	Hrdý et al. (28)
	DSS-induced UC	*Lactobacillus paracasei R3*	Th17/Treg	In DSS-induced colitis in mice, the *L. paracasei R3* strain significantly reduces symptoms and pathological damage while influencing the immune response by altering Th17/Treg cell balance	Huang et al. (226)
	DSS-induced UC	*L. acidophilus*	Th17/Treg	*L. acidophilus* may be a novel treatment for IBD by modulating the balance between Th17 and Treg cells and fibrosis development	Sang et al. (251)
	DSS-induced UC	*L. plantarum 22A-3*	TGF-β1	*L. plantarum 22A-3*-induced TGF-1 production from intestinal epithelial cells enhanced CD103+ DC and Foxp3+ in mice, Treg differentiation, and colitis healing	Lamubol et al. (252)

AIEC, adherent-invasive *Escherichia coli*; COX-2, cyclooxygenase-2; DC, dendritic cell; DSS, dextran sulfate sodium; IL, interleukin; JAK/STAT, Janus kinase/signal transducer and activator of transcription; IFN-γ, interferon-gamma; MAPK, mitogen-activated protein kinase; mRNA, messenger RNA; NF-κB, nuclear factor-κB; MPO, myeloperoxidase; TGF, transforming growth factor beta; TLR, toll-like receptor; TNBS, trinitrobenzene sulfonic acid; TNF-α, tumor necrosis factor alpha; UC, ulcerative colitis.

### 4.2 The role of probiotics in the modulation of MAPK/NF-κB signaling in UC

Chen et al. investigated the impact of *Limosilactobacillus fermentum ZS40* (ZS40) on DSS-induced ulcerative colitis in mice (62). In their research, ZS40 was shown to drastically reduce the production of cytokines that cause inflammation, including IL-1 β, IL-6, and TNF-α, while increasing the synthesis of anti-inflammatory cytokine IL-10 (62). Studies have shown that NF-κB is one of the downstream components in the MAPK signal transduction pathway (159). As a result, activating the MAPK signaling pathway might indirectly promote the NF-κB signaling pathway, generating inflammatory mediators. In conclusion, targeting the NF-κB and MAPK signaling pathways to reduce the expression of inflammation-related genes and proteins, such as IL-6 and TNF-α, represents an effective strategy for treating colitis (62). The study conducted by Chen et al. also assessed the effect of ZS40 on NF-B and MAPK activation. ZS40 might reduce the relative expression of NF-Bp65, IL-6, and TNF-α mRNA and protein while increasing the expression of IB- mRNA and protein. It may also suppress the p38 and JNK1/2 mRNA expression and p38, p-p38, JNK1/2, and p-JNK1/2 protein. Limiting the activation of the NF-κB and MAPK pathways reduces inflammation (62). In summary, the strain can effectively alleviate colitis symptoms in mice and thus can be used to prevent colitis.

Shi and colleagues investigated the therapeutic efficacy of *L. plantarum* subsp. *plantarum SC-5* (SC-5) on DSS-induced colitis in C57BL/6 J mice in another investigation (160). This research shows that SC-5 significantly reduced diarrhea, bloody stools, colon shortening, and weight loss caused by DSS in mice (160). SC-5 increased the stability of the gastrointestinal mucosal barrier and lowered permeability in the gut, which protected mice against DSS-induced colitis (160). A rise in proinflammatory molecules is a crucial indicator that organisms resist inflammation. Proinflammatory cytokines are often significantly elevated in the blood and colon tissue of IBD patients (161). They tested this concept using ELISA and discovered that IL-1β, IL-6, and TNF-α were significantly induced in the colon tissue of DSS-induced colitis mice, indicating a potent anti-inflammatory mechanism. Nevertheless, the concentrations of IL-1β, IL-6, and TNF-α in the colon tissue of mice administered SC-5 decreased, suggesting that the body’s inflammation reaction was reduced (160). They employed Western Blot to evaluate the proteins expression levels of the NF-κB and MAPK signaling pathways to investigate the unique anti-inflammatory mechanism of SC-5. They discovered that SC-5 prevented the induction of the NF-κB pathway by down-regulating the expression levels of p-p65, p-IκB (160). Comparably, SC-5 suppressed MAPK signaling pathway activity by downregulating p-p38, p-JNK, and p-ERK (162), explaining how SC-5 acts as an anti-inflammatory in the body. Finally, SC-5 might shield mice from DSS-induced colitis by suppressing NF-κB and MAPK signaling pathways, enhancing tight junction proteins, enhancing the integrity of the gastrointestinal mucosal barrier, and maintaining gut microbiota configuration balance. It is a probiotic option with promising prospects and potential for development into a unique probiotic therapy for preventing and treating IBD.

### 4.3 The role of probiotics in the modulation of the TLR signaling in UC

By applying TLR-4 mutant (^lps−/lps-^) mice, Chung and colleagues conducted the first investigation to examine the impact of probiotics on the progression of experimental colitis. Mice are deficient in TLR-4, and wild-type (WT) mice were treated with 2% DSS with or without *Lacticaseibacillus casei* (*L. casei*) (163). Evidence shows that TLR-4, at least in certain strains of mice, has a role in the progression of severe diseases in the DSS model (164). Both TLR-4 ^lps−/lps-^ and WT mice treatments with *L. casei* showed a significant reduction in the clinical and histopathological manifestations of DSS colitis (30). MPO activity and IL-12p40 concentrations were also reduced in TLR-4 ^lps−/lps-^ animals that had been primed with DSS (163).

On the contrary, pretreatment TLR-4 ^lps−/lps-^ mice exhibited substantially elevated mRNA and protein expressions of TGF-β and IL-10. TLR-4’s precise function in intestinal inflammation is still up for debate. Rakoff-Nahoum and colleagues recently revealed that TLR-4 plays a significant role in preserving intestinal epithelial homeostasis and defense against direct epithelial damage, even though most research only indicated a function for TLR-4 in inflammatory responses. According to their findings, animals lacking MyD88, which affects TLR signaling, are more prone to intestinal epithelium injury than WT mice. They contended that intestinal epithelial cells’ constitutive TLR signaling causes the synthesis of tissue-protective molecules such as TNF, IL-6, and keratinocyte-derived chemokine-1 (165). Additionally, according to Araki and colleagues, MyD88-deficient animals given DSS developed severe colitis unexpectedly (166). These two research studies often employed TLR- ^4−/−^ or Myd88 ^−/−^ mice, which Dr. Akira donated and which suppressed all TLR signaling.

In contrast to those animals, the TLR-4 ^lps−/lps-^ mice employed in the Chung et al. research are a subset of TLR-4 mutant mice in which TLR signaling is only partially inhibited. They validated a missense mutation in the TLR-4 gene’s third exon, which indicated that proline would be changed to histidine at position 712 of the polypeptide chain. Combining these findings, it is plausible that partial suppression of TLR-4 signaling may be helpful in certain instances of acute epithelium damage, while total inhibition of TLR signaling may do more harm than good. The findings imply that *L. casei* prevents the onset of acute DSS-induced colitis and that this impact primarily depends on TLR-4 status (163). In inadequate TLR-4 complex signaling, *L. casei* modifies the production of inflammatory mediators and decreases neutrophilic recruitment (163).

The underpinning protective functions of *L. plantarum L15* in the amelioration of DSS-induced mice model of colitis were examined by Yu et al. in recent research (30). Previous studies have shown that colonic tissues exposed to DSS have considerably higher levels of cytokines that promote inflammation, such as TNF-α, IL-12, and IL-1β (167, 168). Lactobacillus species have been shown to suppress these cytokines, hence decreasing their proinflammatory effects (169, 170). According to the study’s findings, the DSS group had considerably greater TNF-α, IL-1β, and IL-12 levels than the control group, but high-dose *L. plantarum* L15 treatment completely rectified these abnormalities. Furthermore, this probiotic administration dramatically boosted cytokine levels that prevent inflammation of IL-10 (30). Ning and colleagues earlier hypothesized that LPS actions cause the TLR4 receptor to be activated and that the latter has been associated with low-grade chronic inflammatory disorders (171). Additionally, it has been demonstrated that the TLR4/NF-κB complex, which is created following the effective interaction of the TLR4 receptor with LPS, activates the release of mediators of inflammation (172). It has been indicated that the NF-κB transcription factor activates crucial genes that participate in immunity. These mechanisms and pathways ultimately lead to the onset of UC (173, 174). The expression of TLR4, p-p65, and p-IκB was higher in the DSS group than in the control group in a study conducted by Yu et al., but this tendency was stopped by high doses of *L. plantarum* L15 treatment (30). Additionally, it has been observed that injection of DSS activates TLR4 after it has been upregulated following intestinal assessment of IBD patients (175, 176). This finding suggests that supplementing UC mice with high dosages of *L. plantarum* L15 favorably controlled their gut microbiota. This was shown by a decrease in LPS concentrations, which in turn inhibited the triggering of the TLR4-NF-κB signaling pathway. According to these results, *L. plantarum* L15 may be useful as a probiotic for treating UC since it may better regulate the gut microbiota and lessen an inflammatory response.

Similar to this research, Xia and colleagues looked at how *L. plantarum* AR113 affected the severity of DSS-induced colitis in a different investigation (177). The findings of this investigation demonstrated that the *L. plantarum* AR113 upregulated the expression of IL-10 while concomitantly reducing the production of TNF-α, IL-1β, and IL-6 (177). Recent studies have shown that oxidative stress is a crucial factor in the emergence of colitis (178). According to Xia and colleagues, *L. plantarum* AR113 has an exceptional capacity for antioxidants and may significantly promote the activity of antioxidant enzymes (177). Under normal circumstances, NF-κB is inactive because it is firmly bound to IκB. However, when triggered through the MyD88-dependent pathway, NF-κB becomes quickly active, increasing the transcription of downstream genes involved in inflammatory responses (179). In this research, intervention with *L. plantarum* AR113 or *L. casei AR342* inhibited TLR4/MyD88 signaling and subsequent NF-κB activation (177). An inducible enzyme called HO-1 may reduce inflammatory reactions (180). HO-1 acts as a negative regulator by blocking NF-κB activation, reducing TLR4 expression and the production of inflammatory mediators (181). In conclusion, the research by Xia and colleagues revealed that *L. plantarum* AR113 and *L. casei AR342* administrations might significantly elevate HO-1 expression, suggesting that some probiotics might prevent DSS-induced colitis via modifying the HO-1/TLR4/NF-κB pathway in mice colonic tissues (177).

Since the TLR4/Myd88/NF-κB axis signaling pathway is vital in the regulation of inflammatory reactions, Li et al. suggested that it may be responsible for the protective action of *Lactobacillus rhamnosus* GG effector protein HM0539 (68). The findings of this investigation demonstrated that HM0539 substantially mediated its curative properties by lowering the level of inflammatory responses. They might have expected HM0539 to act as an agent of a well-known therapeutic method for curing IBD according to their findings of the impact of HM0539 in LPS-induced RAW 264.7 macrophages and DSS-induced murine colitis (68). This research showed that the lower MyD88 level might be caused by an HM0539-induced reduction in TLR4 expression, which would block distal NF-κB activation and inflammatory mediators that promote inflammation and lessen the inflammatory reactions brought on by LPS (68). It is still necessary to conduct and confirm further research on the effects of HM0539 on IBD.

Wang and colleagues recently investigated the role of *Bifidobacterium animalis* in DSS-induced colitis (182). According to the study, *B. animalis* substantially reduced spleen weight loss, disease activity index (DAI) score, weight loss, colon shortening, MPO activity, and colon tissue injury (68). Furthermore, *B. animalis* markedly boosted the amount of anti-inflammatory cytokine while dramatically reducing the level of cytokines that promote inflammation (68). The mRNA levels of TLR4, MyD88, and NF-ĸB in the DSS-induced colitis group of this study were significantly higher than the normal control group. Notably, treatment significantly reduced these changes (182). Overall, this research shows that *B. animalis* may maintain intestinal barrier function, regulate inflammatory cytokines, prevent colitis caused by DSS in mice, block TLR4/MYD88/NF-κB activation, and modify the particular gut microbiota (182). This study provides a theoretical framework for further research into using probiotics to treat colitis.

In the current investigation, Huan Kui Le (HKL) suspension and *Lactobacillus acidophilus* (*L. acidophilus*) were investigated in a UC rat model by Aximujiang and colleagues (183). Using 16S rRNA sequencing, immunohistochemistry, ELISA techniques in the colon, and untargeted metabolomics profiling in serum, they investigated the mode of action of this combination therapy (183). The findings showed that IL-12, IFN-γ, IL-6, IL-17, TLR4, and TLR9 expression levels were elevated in the UC group, while TGF-β protein expression levels decreased (183). Colonic epithelial cells seldom express the TLR9 protein, although mucosal infiltrating cells perform, and TLR9 is upregulated throughout inflammatory conditions (184, 185), consistent with Aximujiang et al. results (183). Significant downregulation of TLR4 and TLR9 expression was observed in the Lac and HKL-treated group (183). While dendritic cells (DCs) react to symbiotic bacterial DNA through TLR9 signaling to prevent Treg development in the intestinal tract, pathogenic bacterial DNA stimulates TLR9 expression *in vitro* (186). These findings show that the group treated with Lac and HKL had decreased Treg cell differentiation and TLR9 protein expression, elevated TGF-β and IL-10 protein expression, and healed colon mucosal damage while reestablishing the intestinal microflora balance (183). According to prior research, Lac and HKL treatment raised the number of Treg cells and lowered the number of Th17 cells in the blood of UC rats (183). They argue that probiotics and TCM are innovative approaches for treating UC that alter the gut microbiota and its metabolites, TLR9, and cytokines in several pathways.

### 4.4 The role of probiotics in the modulation of the TLR/MyD88/NF-κB signaling in UC

Wang and colleagues examined the impact of VSL#3, a probiotic, on NF-κB and TNF-α in rats with colitis. They explored the relationship between this impact and the TLR4-NF-κB signal pathway (187). In their study, the levels of TLR4 and NF-κB p65 protein, TLR4, NF-κB, and TNF-α mRNA, as well as the concentration of TNF-α, were all considerably higher in the model and the therapy groups than in the control group. In contrast, the expression and levels of the treatment group were significantly lower than the model group, and the level gradually decreased during the treatment period. These results imply that VSL#3 may cure colitis by decreasing TNF-α expression by blocking the TLR4-NF-κB signal pathway (187). The study observed a strong correlation between TLR4 and NF-κB with TNF-α mRNA in the group of rats undergoing probiotic VSL#3 therapy. This suggests that the TLR4-NF-κB signal pathway is closely associated with changes in inflammatory mediators. Because PCR is the best technique for quantitative detection and is very representative, the findings of PCR were employed for analysis in this research. As upstream components of the TLR4-NF-κB signal pathway, TLR4 and NF-κB mediate and control this route’s functionality (188). The findings of a study examining NF-κB and TLR4 in individuals with colitis are consistent with the research conducted by Wang et al. (189). In conclusion, probiotic VSL#3 is anticipated to be a first-choice medication in managing colitis because it reduces the production of NF-κB and TNF-α in rats with colitis via the TLR4-NF-κB signal pathway.

In earlier research, a new soluble protein from *L. rhamnosus GG* (LGG), HM0539, had considerable protective benefits against mouse colitis. However, no precise, specific mechanism for this action was presented (68). In a different research, Li et al. proposed that HM0539’s anti-inflammatory properties may result from manipulating the TLR4/Myd88/NF-κB axis signaling pathway. This vital route is heavily involved in regulating inflammatory reactions (68). The findings of the present investigation demonstrated that HM0539 substantially mediated its protective effects by lowering the inflammatory process. In light of Li et al.’s findings of HM0539’s effects on DSS-induced murine colitis and LPS-induced RAW 264.7 macrophages, they could anticipate HM0539 to act as an agent of a well-known therapeutic approach for managing IBD (68). The body’s most significant concentration of macrophages is found in the gastrointestinal mucosa. Within the intestinal lamina propria and epithelial layer, macrophages are highly abundant but may be found across the whole gastrointestinal mucosa (190, 191). Macrophages could potentially be investigated as possible targets for immunological modulation of probiotics. The generation of prostaglandin E2 (PGE2) and NO was suppressed as a consequence of HM0539’s ability to limit the expression of cyclooxygenase-2 (COX-2) and iNOS by reducing the activation of their respective promoters (68). They also discovered that HM0539 has a part in modulating the immune response, as shown by the consequent reduction of inflammatory mediators such as IL-1β, TNF-α, IL-6, and IL-18 released by macrophages (68). Li et al. discovered that the potential impact of HM0539 on the initiation of distal NF-κB could be attributed to its ability to reduce TLR4 activation and impede MyD88 transduction (68). The anti-inflammatory effects of HM0539 were clearly abolished by the upregulation of TLR4 or MyD88, although the compound still had some impact on LPS-induced inflammatory processes (68). Finally, HM0539 was demonstrated to be a promising anti-inflammatory drug, at least in part, by its decreased levels of the TLR4-MyD88 axis and of the downstream MyD88-dependent activated NF-κB signaling, and therefore it may be evaluated as a viable treatment alternative for IBD.

The research project conducted by Yu et al. aimed to elucidate the mechanisms underlying the capacity of *L. plantarum* to maintain the stability of UC (30). First, 15 strains of *L. plantarum* were analyzed for their probiotic potential based on their resistance to the simulated human gastrointestinal transit and adhesion. Second, the inflammatory reaction of specific strains to the LPS-induced Caco-2 cells was quantified. Finally, the effectively screened *in vitro* strain *L. plantarum L15* was evaluated for its positive effects and potential action mechanisms using an *in vivo* mouse model caused by DSS (30). To assess UC conditions, DAI and MPO are often employed (192, 193). The DAI and MPO considerably rose in the DSS group in the current research, showing that the UC model was effectively developed. The two indices were reversed by supplementing with a high dosage of *L. plantarum L15*. These findings demonstrated that mice treated with DSS might successfully restore their physiological abnormalities by supplementing with *L. plantarum L15* (68). Ning and colleagues previously hypothesized that LPS activities cause the TLR4 receptor to be activated and that the latter has been associated with low-grade chronic inflammatory disorders (171). The TLR4/NF-κB complex, which is created when the TLR4 receptor and LPS successfully connect to each other, activates the release of inflammatory mediators (172). The TLR4/NF-κB complex, which is created when the TLR4 receptor and LPS properly link to each other, stimulates the release of inflammatory cytokines (172). It has been shown that the NF-κB transcription factor activates crucial genes participating in the generation of proinflammatory cytokines, eventually impairing the body’s defenses. These mechanisms and pathways ultimately lead to the onset of UC (174). TLR4, p-p65, and p-IB expression increased in the DSS group compared to the control group in the current study; however, this trend was halted by high-dose *L. plantarum L15* administration. Additionally, TLR4 increased levels have been observed throughout the gastrointestinal examination of IBD patients; its activation by DSS administration has been documented (176). In conclusion, their study’s *in vitro* and *in vivo* findings showed that L. plantarum L15 had excellent intestinal transit tolerance, adhesion properties, and a strong ability to reduce proinflammatory activity. Additionally, large doses of *L. plantarum L15* administration favorably controlled the gut flora of UC mice, as shown by the downregulation of LPS levels, which, in turn, inhibited the triggering of the TLR4-NF-κB signaling pathway.

Xia and colleagues also investigated the underpinning molecular function of *Lactobacillus plantarum* AR113 against DSS-induced colitis (177). Compared to other probiotic strains, *L. plantarum AR113* and *L. casei AR342* are more effective in treating colitis brought on by DSS and maintaining the integrity of the intestinal barrier. More significantly, these two strains could control the TLR4/MyD88/NF-κB inflammatory signaling pathway well. *L. plantarum AR113* may also change the dysbiosis of the gut microbiota and prevent the spread of potentially harmful pathogens (177, 178). Recent studies have shown oxidative stress as a key contributor to the etiology and progression of colitis. *L. plantarum AR113* has the potential to both start and worsen inflammation. Antioxidant enzyme activity might be significantly boosted by the suggested probiotic *L. plantarum AR113.* The property of *L. plantarum AR113’s* antioxidant capacity was why they conducted this research (176).

The obtained results showed that treatments with *L. plantarum AR113* and *L. casei AR342* inhibited TLR4/MyD88 signaling and the subsequent activation of NF-κB (177). An inducible enzyme, HO-1, may reduce inflammatory reactions (180). By blocking the NF-κB activation, HO-1 acts as a negative regulator, reducing TLR4 expression and preventing excessive cytokine production associated with inflammation (181). Treatment with either *L. plantarum AR113* or *L. casei AR342* dramatically upregulated HO-1 expression, as shown in research by Xia and colleagues, suggesting that these particular probiotics safeguard against DSS-induced colitis through regulating the HO-1/TLR4/NF-κB pathway in the tissues of the mouse colon (177). In conclusion, eight probiotics were tested for their potential to alleviate symptoms of DSS-induced colitis in C57BL/6 J mice. Intestinal administration of *L. plantarum AR113* and *L. casei AR342* was reported by Xia and colleagues to modulate the immunological response, preserve the epithelial barrier, and reduce gut microbiota dysbiosis. Increasing HO-1 expression with *L. plantarum AR113* and *L. casei AR342* supplementation has the potential to reduce the production of the inflammatory mediators TNF-α, IL-1β, and IL-6 by blocking the TLR4/MyD88/NF-κB pathway. Their results will inform the future research showing how probiotics genuinely improve human health.

### 4.5 The role of probiotics in the modulation of the NLRP-3 signaling in UC

A common probiotic, *Enterococcus faecalis* (*E. faecalis*), was tested to see whether it may inhibit the NLRP3 inflammasome and so prevent colitis and colitis-associated colorectal cancer (CRC) in research by Chung et al. (194). The researchers investigated how heat-destructed *E. faecalis* cells affected the induction of the NLRP3 inflammasome in THP-1-derived macrophages. The induction of the NLRP3 inflammasome in macrophages in reaction to either fecal content or commensal microorganisms, *Proteus mirabilis* or *Escherichia coli*, may be inhibited by pretreatment with *E. faecalis* or NLRP3 siRNA, as seen by a decrease in caspase-1 activation and IL-1β maturation. *E. faecalis* impedes the process of phagocytosis, which is an essential step for the complete activation of the NLRP3 inflammasome. *In vivo* studies on animals have demonstrated that *E. faecalis* confers protection against DSS-induced colitis and CRC in wild-type mice by mitigating intestinal inflammation (194). In addition, *E. faecalis* does not protect NLRP3 knockout mice from developing colitis when exposed to DSS. The research by Chung and colleagues suggests that using inactivated *E. faecalis* as a probiotic to reduce NLRP3-mediated colitis and inflammation-associated colon carcinogenesis is a feasible and safe option (194).

Dou and colleagues analyzed the probable mechanism of action of *L. casei ATCC 393* and its metabolites on DSS-induced mice colitis (195). Studies have shown that *L. casei ATCC 393* substantially reduces intestinal barrier malfunction caused by enterotoxigenic *E. coli K88* (196). These findings demonstrate that oral treatment of *L. casei ATCC 393* and its metabolites significantly reversed DSS-induced weight loss and reduced DAI, colon length, and villus height of colon tissue in mice. The levels of gene expression of occludin, ZO-1, and claudin-1 elevated, and the expression of NLRP-3, Caspase-1, IL-1β, and IL-18 were decreased compared to the DSS-induced model group. Metabolites from *L. casei ATCC 393* also significantly impeded the penetration of immune cells into the intestinal mucosa. Dysbiosis of the gut microbiota caused by DSS was also efficiently treated by *L. casei ATCC 393* and its metabolites (195). In conclusion, the data presented here indicated that *L. casei ATCC 393* and its metabolites reduced the NLRP3-(Caspase-1)/IL-1β signaling pathway-mediated ulcerative inflammatory reaction in C57BL/6 mice after DSS administration.

The latest investigation by Qu and colleagues explores the role of *Akkermansia muciniphila* (*A. muciniphila*) in the context of acute colitis (29), acknowledging that its commercial use as an inactivated product differs from the live form studied. Researchers have shown that *A. muciniphila* numbers are lower in those with UC (197); results from the GMrepo database agree with those found by Qu et al. More research is needed to determine why and how the number of *A. muciniphila* cells in UC patients declines. One hypothesis is that the reduction of the mucus layer on colonocytes in UC patients leads to decreased availability of polysaccharides, which are essential for the growth of *A. muciniphila* (198–200). This decline in mucus may impair the ability of *A. muciniphila* to thrive, contributing to its reduced numbers in the gut.

Taking supplements with *A. muciniphila* dramatically reduced symptoms of acute colitis in mice (29). *A. muciniphila* was thought to regenerate the mucus layer and preserve intestinal function (201). *A. muciniphila* has been shown to improve the symptoms of DSS-induced UC by increasing the efficiency of intestinal integrity (202). In addition, *A. muciniphila* suppressed the production of IL-1β, MCP-1, and IL-6, all released by locally activated cells associated with inflammation (29). These findings suggested that *A. muciniphila* might serve as a prophylactic against experimental colitis. Some investigations have shown *A. muciniphila* to have a mixed impact on colitis. In mice infected with *Salmonella enterica* serovar Typhimurium, *A. muciniphila* increased inflammation in the intestines (203). In IL-10−/− mice, the overexpression of *A. muciniphila* caused by NLRP6 deficiency exacerbates intestinal tract inflammation (204). Nonetheless, *A. muciniphila* is not pathogenic on its own (205). The role of *A. muciniphila* could potentially be altered by its interactions with other bacteria or compounds produced by other commensals (206). Supplementation with *A. muciniphila* increased NLRP3 expression *in vitro* and *in vivo*, as shown in research by Qu and colleagues (29). In addition, the protective role of *A. muciniphila* in colitis was abolished in mice lacking the NLRP3 gene (29). IL-1β and IL-18, both of which are known to protect against infections and environmental stimuli, are released in response to NLRP3 activation (207, 208). Mice with NLRP3 deficiency are more likely to develop experimental colitis (209–211); this vulnerability might be reversed by administering exogenous IL-1β or IL-18 (207). In light of this, Qu and colleagues hypothesize that NLRP3 expression is linked to colon epithelial healing during gut inflammation. It was concluded that the upregulation of NLRP3 in the colon tissues of UC patients was protective. Based on these findings, it seems that probiotic-based treatment for colitis benefits from NLRP3’s protective properties. However, the precise mechanism of its regulation warrants more investigation.

### 4.6 The role of probiotics in the modulation of the JAK/STAT signaling in UC

Through JAK/STAT and other signaling pathways, probiotics have been shown to positively influence inflammatory processes and immunological modulation (212–215). Overall, this research suggested that a cocktail of *Lactobacillus* spp. had an anti-inflammatory impact on HT-29 cells by influencing the JAK/STAT and NF-κB signaling pathways. They concluded that Lactobacillus spp., taken as a dietary supplement, may help prevent and treat inflammatory disorders such as IBD (212). In this investigation, the JAK/STAT pathway yielded contrasting outcomes. It can be inferred that Lactobacillus spp. has the potential to either inhibit or enhance expression. The phenotypic observations and the anti-inflammatory properties of probiotics are consistent with the findings mentioned above. The study conducted by Rohani et al. revealed a general reduction in the expression of specific STATs, namely STAT3 and STAT6 (212).

In most cases, inhibiting this pathway results in significant reductions in inflammation. Many investigations have shown that suppressing STAT3 expression, for instance, may lead to a drop in IL-6 and, by extension, contribute to calming down the level of inflammation (57, 216). Another STAT whose phosphorylation is elevated in UC patients is STAT6. Limited colitis has been seen in STAT 6^−/−^ animals, suggesting that STAT6 deficiency might be beneficial for enhancing IBD (217). Remarkable evidence on the regulation of inflammation was also found in the JAKs research by Rohani and others. All three JAKs (JAK1, JAK2, and JAK3) were declining (212). In light of this, various studies have considered the possibility of inhibiting IBD by targeting JAKs (218). In summary, determining how probiotics modify and decrease inflammation may need a more in-depth understanding of the specific molecular effects of *Lactobacillus* spp. on signaling pathways. Whether or not probiotics may be utilized as a preservative or a treatment is one of the most pressing questions in this area. Patients with IBD may benefit significantly from discovering the simplest means of both preventing and treating their symptoms. The findings may imply that these probiotic strains might prevent or mitigate the severity of IBD since they utilize *Lactobacillus* spp. as pre-, post-, and cotreatment and observe its beneficial impact on all three variations.

Recent research by Aghamohammad and colleagues compared the probiotics *Lactobacillus* spp., Bifidobacterium spp., and a combination of the two for their ability to inhibit inflammatory signaling through the JAK/STAT pathway (219). The NF-κB pathway genes JAK, TIRAP, IRAK4, NEMO, and RIP decreased by the probiotic cocktail compared to cells treated with sonicate pathogens. After receiving probiotic medication, there was some variation in the expression of STAT genes. After receiving probiotic medication, production of IL-6 and IL-1β was reduced (219). Probiotic therapies were associated with a considerable reduction in inflammatory genes, as measured by the NF-κB signaling pathway. The effects of probiotic strains on HT-29 cells were opposite to those of the pathogen when the pathogen was sonicated. However, their probiotic strains, whether used alone or in combination, reduced the mRNA level of the examined inflammatory genes, while sonicated pathogens increased their expression. These findings might have a molecular basis, and this may explain it, according to prior research (220), in which Aghamohammad and colleagues have shown that administration of these particular probiotics significantly reduced IBD-induced inflammatory reactions in mice (219). They found contradictory data about STAT gene expression in the present investigation. Different probiotic treatments either increased or decreased the expression of some genes.

The expression levels of STAT1, 2, and 4 were observed to be upregulated and downregulated, whereas STAT3 and STAT5 exhibited a notable increase in expression. Conversely, the expression of STAT6 was observed to be decreased (219). They also found that probiotics reduced JAK expression by inhibiting the activity of JAK genes (219). According to research by Aghamohammad and colleagues, probiotics have an anti-inflammatory effect comparable to that of JAK inhibitors such as JAKinibs, agents that target JAK by decreasing JAK expression (221). Gene expression was significantly influenced by *Lactobacillus* spp., Bifidobacterium spp., and Lac/Bif, all of which played unique roles in each gene (219). This probiotic mixture inhibited inflammatory responses in HT-29 cells by downregulating the JAK/STAT and NF-κB pathways, and for this reason, taking *Lactobacillus* spp. and *Bifidobacterium* spp. Probiotics as dietary supplements may help lessen the risk of inflammation-related disorders, such as IBD.

### 4.7 The role of probiotics in the modulation of the Wnt/β-catenin signaling in UC

You and colleagues found that UC tissues had different Wnt ligand and receptor expression than controls (131); however, the alterations did not correlate with any noticeable increase or decrease in disease activity, and no following research was able to confirm these initial results (132). However, chronic IBD and animal models both activate the Wnt/β-catenin pathway (133). Mucosal healing in IBD is facilitated by epithelial TNF receptor signaling (134). These incongruous insights should not be seen as a surprise. High compartmentalization of Wnt ligand expression in the gut (135). This issue of differentiation and their prior participation provide a comprehensive assessment of non-epithelial Wnt sources in the gut and their function in tissue homeostasis (136, 137). Recent advances in the area also imply that inflammatory compounds are in charge of maintaining and regenerating the epithelium throughout colitis. For UC patients, targeting Wnt/β-catenin might hold promise for attaining the best results. The Wnt/β-catenin pathway has been demonstrated to be modulated by probiotics, which may help with UC symptoms (222).

The effects of probiotics coupled with sulfasalazine (salicylazosulfapyridine [SASP]) on the expression of the Wnt/β-catenin signaling pathway in rats with UC were examined by Dong and colleagues in recent research (65). The Wnt/β-catenin signaling pathway is believed to be essential for the growth and differentiation of intestinal epithelial cells (223), which is active in the epithelial cells of individuals with UC and is essential for the proliferation and differentiation of intestinal epithelial cells. The degree of histological differentiation of colon cancer may be affected by colitis, which can induce nuclear translocation of β-catenin. In this research, Dong et al. discovered a unique effect of probiotics with SASP on the UC mouse model (223). With the treatment of probiotics in conjunction with SASP, the ulcer area significantly decreased in the model group, and disease-related clinical symptoms such as diarrhea and rectal hemorrhage decreased. These results indicated the cytoprotective and anti-ulcer abilities of the probiotics (223). Multiple factors, including ligand/receptor interactions, β-catenin translocation, and transcriptional activation, contribute to the intricate modulation of the Wnt signaling pathway (224). According to research by Dong and colleagues, Wnt ligand expression was shown to be increased in the mucosa of UC rats. Both lamina propria and epithelial cells showed an increase in these ligands as a part of the damage response (223). Overall, this research demonstrated that the Wnt signaling pathway’s excessive stimulation encourages the emergence of UC. Probiotics coupled with SASP therapy may inhibit the Wnt pathway’s aberrant excessive activation and provide anti-ulcer benefits (223). These findings may be implemented as a complement to existing research and as a springboard for developing novel approaches to the management of UC.

Another study found that using a DSS-induced mouse model of colitis, VSL#3 inhibits tumorigenesis in mice and cells via modulating the inflammatory and Wnt/β-catenin route. Since it is challenging to evaluate probiotics’ impact on UC-associated tumorigenesis clinically, studies of this condition often employ animal models. Previous research has shown that combining azoxymethane (AOM) and DSS may rapidly create a UC-associated tumorigenesis model (222). Though preliminary evidence from mice models suggests that VSL#3 suppresses tumorigenesis in UC, definitive results are yet to be obtained. VSL#3 protects against cancer linked to UC, although how it does so is not well understood now (222). To confirm the effectiveness of VSL#3 in suppressing UC-associated tumorigenesis and examine the precise mechanism of action, Li et al. employed a mouse model of AOM/DSS-induced UC-associated tumorigenesis. They proceeded deeper to investigate how probiotics affect the Wnt/β-catenin pathway at the human cellular level, taking into account the distinctions between mice and humans. Li et al. chose Bifidobacterium to investigate the impact and mechanisms of VSL#3 on UC-associated tumorigenesis in mice and cells since it is challenging to grow numerous probiotics concurrently (222). VSL#3 supplementation and co-culturing cells with Bifidobacterium might decrease the proinflammatory components TNF-α and IL-6, inhibit NF-κB transcriptional activity, and then downregulate the Wnt/β-catenin pathway, thus preventing the development from inflammatory processes to tumor formation, as shown in this study (222). In sum, VSL#3 has shown promise as a therapeutic agent for treating UC-related tumorigenesis.

### 4.8 The role of probiotics in the modulation of IL-23/IL-17 signaling in UC

Park et al. found that *L. acidophilus* reduced Th17-derived cytokines that promote inflammation (IL-6, TNF-α, IL-1β, and IL-17) in DSS-induced mouse models of colitis (225). Additional findings from this research showed that *L. acidophilus* administration directly promoted Treg cells and IL-10 production while suppressing IL-17 production in splenocytes when tested *in vitro* (225). According to the findings of Park et al., *L. acidophilus* has the potential to be a new therapy for IBD by adjusting the ratio of Th17 to Treg cells. Hrdý et al. tested the *L. acidophilus* strain BIO5768 for its modulatory effects on the immune system in both basal and acute inflammatory conditions (28). The findings show that BIO5768 may stimulate the production of IL-17 target genes such as Angiogenin-4, an effect attenuated in IL-17 receptor-null animals. Additionally, the researchers discovered that BIO5768 promoted IL-22 synthesis by type 3 innate lymphoid cells and improved DC function to facilitate IL-17 release by CD4+ T cells (28). In addition, colitis brought on by TNBS and intestinal inflammation brought on by *Citrobacter rodentium* infection were both improved by BIO5768. These results imply that the probiotic *L. acidophilus* strain BIO5768 may be useful in treating IBD (28). In a recent investigation, Huang and colleagues tested *Lactobacillus paracasei R3* (*L.p R3*) against DSS-induced colitis (226). A strain of *L.p R3* with high biofilm-forming potential was isolated from newborn feces for this investigation. According to the research, *L.p R3* may reduce the pathological harm and symptoms of mice with colitis by DSS (226). In DSS-induced colitis, L.p R3 was observed to reduce inflammatory cell infiltration, block Th17, and promote Treg activity, suggesting that it controls the balance of Th17/Treg cells. Notably, *L.p R3* may be used to treat colitis by modulating immunological function (226).

Recently, Chen et al. analyzed *L. acidophilus* function in a DSS-induced mouse model of colitis (57). Consistent with earlier observations, they discovered a substantial upsurge in the production of IL-17, a characteristic cytokine identifying Th17 cells, in cases of colitis (227–229). Additionally, they provide the first proof that *L. acidophilus*, one of the most prevalent naturally occurring residents of the human gut, may reduce the elevation of the proinflammatory cytokine IL-17 in colitis when taken orally. A recent study by Jan et al. shows that oral *L. gasseri* treatment reduces IL-17 induction in allergen-induced airway inflammation in mice and improves their outcome (230). The expression of IL-17 and, to a lesser degree, TNF-α was recently downregulated in collagen-induced arthritic mice after treatment of *L. acidophilus*, as shown by Amdekar et al. (231). Similarly, Chen and colleagues found that *L. acidophilus* reduced TNF-α expression in the colons of mice exposed to colitis (57). According to our earlier findings, *L. acidophilus* may benefit the etiology of colitis due to a reduction in IL-17 expression (232). *L. acidophilus* supplementation might have altered the fates of Th17 cells due to decreased levels of IL-17.

Based on mounting evidence, IL-23 is crucial for sustaining Th17 cell proliferation and survival, while TGF-β1 is crucial for Th17 cell differentiation. Additionally, new research has shown that IBD is associated with increased levels of TGF-β1 and IL-23 expression (233, 234), which promotes the development of inflammation by triggering Th17 differentiation and function. This study provided the first evidence that *L. acidophilus* inhibits the production of TGF-β1 and IL-23 in the colon of animals with colitis (57). These findings suggest that *L. acidophilus* therapy has a beneficial effect via reducing TGF-β1 and IL-23 production, which may influence the Th17 cell population (57). To prove this theory, immunohistochemistry labeling of a Th17 cell-specific marker like IL-17 in colitis treated with and without *L. acidophilus* is required. Chen et al. did not elucidate the molecular mechanisms underlying the inhibitory impact of *L. acidophilus* on IL-23 modulation in their recent study. However, they postulate that the downregulation of TNF-α expression may be accountable for this phenomenon (57). In conclusion, Chen et al. showed that oral administration of *L. acidophilus* suppressed colitis-associated hyper-response of the IL-23/Th17 axis. Specifically, *L. acidophilus* treatment inhibited the secretion of proinflammatory cytokine IL-17 by downregulating IL-23 and TGF*β*1, which are required for Th17 cell differentiation and stabilization. These findings indicate that the therapeutic role of *L. acidophilus* in IBD treatment, at least in part, involves modulating the IL-23/Th17 immune axis (57).

Leccese and colleagues analyzed the effects of *Lactobacillus* and *Bifidobacterium* strains on the virulence mechanisms of adherent-invasive *E. coli* (AIEC)-LF82 and the subsequent inflammatory reaction associated with the CCR6-CCL20 and IL-23/Th17 axis in patients with CD and UC (67). By interacting with DCs, probiotic bacteria not only reduce levels of proinflammatory polarizing cytokines but also increase levels of the anti-inflammatory cytokine IL-10, hence enhancing DC tolerogenic function (235, 236); however, it is also vital in avoiding the beginning of chronic inflammatory processes. Indeed, DCs play a crucial role in preserving intestinal tolerance and immunological balance. However, they also aggregate in areas of intestinal inflammation, where they induce an induction of bacteria-specific T cell subsets found in the lamina propria, so contributing to the pathophysiology of IBD (237, 238). As more evidence emerges demonstrating the harmful function of the IL-23/Th17 axis in IBD (239, 240), it seems to be evident that suppressing the microbial determinants leading to this detrimental inflammatory reaction in DCs whereas reestablishing the eubiotic structure of the gut microbiota might be an appealing therapeutic approach for IBD (241). The data presented by Leccese and colleagues show for the first time that LF82 may provoke a different production of polarizing cytokines, which leads to different effector Th-cell subsets polarization based on DCs origin. For example, after 24 h of infection, CD-derived monocyte-derived dendritic cells (MoDC) produce significantly more IL-23 than UC-derived MoDC, boosting the expansion of pathogenic Th17 cells (67). Collectively, their data show that the *Lactobacillus* and *Bifidobacterium* strains investigated here inhibit the absorption and persistence of LF82 within DCs, and that they all inhibit the IL-23/Th17 axis, to a similar extent as the anti-inflammatory drug 6MP, as well as promoting the generation of the cytokine that inhibits inflammation, IL-10, in both healthy donors and UC patients. Probiotic strains, except for the *B. breve* Bbr8 strain, are substantially less efficient in influencing the LF82-induced inflammatory reaction and hindering the production of the polarizing IL-1 and IL-23 cytokines that control the development of pathogenic Th17 cells in CD-derived DCs (242, 243). Overall, the purpose of this research was to examine the impact of different *Lactobacillus* and *Bifidobacterium* strains on the ability of AIEC LF82 to adhere to and remain inside intestinal epithelial cells, macrophages, and DCs. The findings demonstrated that all probiotic strains suppressed IL-8 release, decreased LF82 survival within macrophages, and decreased LF82 adherence and persistence inside intestinal epithelial cells. All probiotic strains could disrupt the IL-23/Th17 axis in UC patients but not in CD patients. The research also discovered that probiotic strains might reduce inflammation brought by AIEC in healthy donors but not in those with IBD. These results imply that studies on immune cells obtained from CD patients may be required to uncover probiotic strains that might benefit CD patients (67).

## 5 Safety and adverse effects of probiotics on UC

It is essential to consider the safety of probiotics and potential adverse effects when used to treat UC. Probiotics are generally recognized as safe (GRAS) by the U.S. Food and Drug Administration (FDA) and the European Food Safety Authority (EFSA) (41). Moreover, probiotics are naturally occurring microorganisms found in the human gut and are part of the normal gut microbiota (48).

However, some reports have been of adverse effects associated with probiotic use, particularly in individuals with compromised immune systems or underlying medical conditions. Probiotics’ most commonly reported adverse effects include gastrointestinal symptoms such as bloating, gas, diarrhea, and abdominal discomfort (244). These symptoms are usually mild and transient and often resolve independently without medical intervention. In particular, some studies have reported an increased risk of adverse effects in UC patients taking probiotics. For example, a systematic review and meta-analysis of randomized controlled trials (RCTs) found that UC patients taking probiotics experienced more adverse events than placebo patients (245). However, the authors noted that the quality of the evidence was low and that further research is needed to determine the safety profile of probiotics in UC patients. It is also important to note that the safety and efficacy of probiotics may vary depending on the strain, dose, and duration of treatment. Therefore, it is essential to consult with a healthcare professional before starting probiotic therapy, especially in individuals with underlying medical conditions. However, some studies have recommended monitoring for symptoms such as fever, abdominal pain, and bloody diarrhea (246).

Additionally, healthcare professionals should be aware of the potential for probiotics to interact with other medications, particularly antibiotics and immunosuppressants (247). While probiotics are designed to promote the growth of beneficial bacteria in the gut, there have been rare reports of infections associated with probiotic use. Infections can occur when probiotics are contaminated with harmful bacteria or when individuals take them with weakened immune systems. In particular, there have been reports of sepsis, endocarditis, and meningitis associated with the use of probiotics in individuals with underlying medical conditions (246). Therefore, individuals with a history of allergies should use probiotics cautiously and consult a healthcare professional before starting probiotic therapy. Probiotics may interact with certain medications, particularly antibiotics and immunosuppressants. For example, some probiotic strains may reduce the efficacy of antibiotics by competing for absorption in the gut (248). There is currently no standardized regulation for the production and labeling of probiotics. Therefore, choosing high-quality probiotic products from reputable manufacturers is vital to ensure safety and efficacy (247).

## 6 Conclusion and perspectives

In summary, the studies reviewed suggest that probiotics hold great potential for preventing and treating UC and other inflammation-related diseases. Probiotics have been found to modulate various signaling pathways, including the NF-κB, NLRP3, JAK/STAT, MAPK/NF-κB, transforming growth factor beta (TGF-β), Wnt/β-catenin, and IL-23/IL-17 pathways, which are essential in the pathogenesis of UC. By influencing these pathways, probiotics can reduce inflammation, promote mucosal healing, enhance the expression of anti-inflammatory molecules, and improve the integrity of the intestinal epithelial barrier. However, it is essential to note that not all probiotics have the same effect on these pathways, and their efficacy may vary depending on the strain, dose, and duration of treatment. Further research is needed to identify the most effective probiotic strains and optimal treatment regimens for UC patients. Of note, the molecular mechanisms underlying the effects of probiotics on inflammatory pathways need to be fully understood. Overall, the findings of these studies suggest that probiotics may provide a safe and effective strategy for attenuating inflammation and preventing UC-associated carcinogenesis.

Further clinical trials are needed to validate these findings and determine the most effective probiotic strains and dosages for UC patients. The use of probiotics as a complementary therapy for UC warrants further exploration and can potentially improve the lives of those suffering from this debilitating disease. There is still much to learn about the use of probiotics in treating UC. While some studies have shown promising results, others have been inconclusive or even contradictory. Therefore, the future research should focus on identifying the most effective strains of bacteria and the optimal dosages and treatment regimens. In addition, studies should be conducted to determine the long-term safety and efficacy of probiotics, as well as their potential to interact with other medications. Ultimately, a deeper understanding of the mechanisms by which probiotics modulate pathogenic signaling pathways in ulcerative colitis will pave the way for developing more targeted and effective therapies for this debilitating disease.

## References

[1] Ng SC Shi HY Hamidi N Underwood FE Tang W Benchimol EI. Worldwide incidence and prevalence of inflammatory bowel disease in the 21st century: a systematic review of population-based studies. Lancet. (2017) 390:2769–78. doi: 10.1016/S0140-6736(17)32448-0 29050646

[2] Zhao M Gönczi L Lakatos PL Burisch J. The burden of inflammatory bowel disease in Europe in 2020. J Crohn's Colitis. (2021) 15:1573–87. doi: 10.1093/ecco-jcc/jjab029, PMID: 33582812

[3] Hugot J-P Chamaillard M Zouali H Lesage S Cézard J-P Belaiche J. Association of NOD2 leucine-rich repeat variants with susceptibility to Crohn's disease. Nature. (2001) 411:599–603. doi: 10.1038/35079107, PMID: 11385576

[4] Petagna L Antonelli A Ganini C Bellato V Campanelli M Divizia A. Pathophysiology of Crohn’s disease inflammation and recurrence. Biol Direct. (2020) 15:23. doi: 10.1186/s13062-020-00280-5, PMID: 33160400 PMC7648997

[5] Torres J Bonovas S Doherty G Kucharzik T Gisbert JP Raine T. ECCO guidelines on therapeutics in Crohn's disease: medical treatment. J Crohn's Colitis. (2020) 14:4–22. doi: 10.1093/ecco-jcc/jjz180 31711158

[6] Collins P Rhodes J. Ulcerative colitis: diagnosis and management. BMJ. (2006) 333:340–3. doi: 10.1136/bmj.333.7563.340, PMID: 16902215 PMC1539087

[7] Sartor RB. Mechanisms of disease: pathogenesis of Crohn's disease and ulcerative colitis. Nat Clin Pract Gastroenterol Hepatol. (2006) 3:390–407. doi: 10.1038/ncpgasthep0528, PMID: 16819502

[8] Kaur A Goggolidou P. Ulcerative colitis: understanding its cellular pathology could provide insights into novel therapies. J Inflamm. (2020) 17:1–8. doi: 10.1186/s12950-020-00246-4 PMC7175540 32336953

[9] Rhen T Cidlowski JA. Antiinflammatory action of glucocorticoids—new mechanisms for old drugs. N Engl J Med. (2005) 353:1711–23. doi: 10.1056/NEJMra050541, PMID: 16236742

[10] Van Dieren JM Kuipers EJ Samsom JN Nieuwenhuis EE Van Der Woude JC. Revisiting the immunomodulators tacrolimus, methotrexate, and mycophenolate mofetil: their mechanisms of action and role in the treatment of IBD. Inflamm Bowel Dis. (2006) 12:311–27. doi: 10.1097/01.MIB.0000209787.19952.53, PMID: 16633053

[11] Ananthakrishnan AN. Environmental risk factors for inflammatory bowel disease. Gastroenterol Hepatol. (2013) 9:367–74. PMID: 23935543 PMC3736793

[12] Satsangi J Jewell D Rosenberg W Bell J. Genetics of inflammatory bowel disease. Gut. (1994) 35:696–700. doi: 10.1136/gut.35.5.696, PMID: 8200569 PMC1374760

[13] Sheehan D Moran C Shanahan F. The microbiota in inflammatory bowel disease. J Gastroenterol. (2015) 50:495–507. doi: 10.1007/s00535-015-1064-1 25808229

[14] Pisani A Rausch P Bang C Ellul S Tabone T Marantidis Cordina C. Dysbiosis in the gut microbiota in patients with inflammatory bowel disease during remission. Microbiol Spectr. (2022) 10:e00616–22. doi: 10.1128/spectrum.00616-22 35532243 PMC9241752

[15] Santana PT Rosas SLB Ribeiro BE Marinho Y De Souza HS. Dysbiosis in inflammatory bowel disease: pathogenic role and potential therapeutic targets. Int J Mol Sci. (2022) 23:3464. doi: 10.3390/ijms23073464, PMID: 35408838 PMC8998182

[16] Shen Z-H Zhu C-X Quan Y-S Yang Z-Y Wu S Luo W-W. Relationship between intestinal microbiota and ulcerative colitis: mechanisms and clinical application of probiotics and fecal microbiota transplantation. World J Gastroenterol. (2018) 24:5–14. doi: 10.3748/wjg.v24.i1.5, PMID: 29358877 PMC5757125

[17] Ahmed J Reddy BS Mølbak L Leser TD Macfie J. Impact of probiotics on colonic microflora in patients with colitis: a prospective double blind randomised crossover study. Int J Surg. (2013) 11:1131–6. doi: 10.1016/j.ijsu.2013.08.019, PMID: 24060951

[18] Degruttola AK Low D Mizoguchi A Mizoguchi E. Current understanding of dysbiosis in disease in human and animal models. Inflamm Bowel Dis. (2016) 22:1137–50. doi: 10.1097/MIB.0000000000000750, PMID: 27070911 PMC4838534

[19] Lobionda S Sittipo P Kwon HY Lee YK. The role of gut microbiota in intestinal inflammation with respect to diet and extrinsic stressors. Microorganisms. (2019) 7:271. doi: 10.3390/microorganisms7080271, PMID: 31430948 PMC6722800

[20] El-Saadony MT Alagawany M Patra AK Kar I Tiwari R Dawood MA. The functionality of probiotics in aquaculture: An overview. Fish Shellfish Immunol. (2021) 117:36–52. doi: 10.1016/j.fsi.2021.07.007, PMID: 34274422

[21] Jadhav A Jagtap S Vyavahare S Sharbidre A Kunchiraman B. Reviewing the potential of probiotics, prebiotics and synbiotics: advancements in treatment of ulcerative colitis. Front Cell Infect Microbiol. (2023) 13:1268041. doi: 10.3389/fcimb.2023.1268041, PMID: 38145046 PMC10739422

[22] Ma Y Yang D Huang J Liu K Liu H Wu H. Probiotics for inflammatory bowel disease: is there sufficient evidence? Open Life Sci. (2024) 19:20220821. doi: 10.1515/biol-2022-0821, PMID: 38585636 PMC10998680

[23] Maftei N-M Raileanu CR Balta AA Ambrose L Boev M Marin DB. The potential impact of probiotics on human health: An update on their health-promoting properties. Microorganisms. (2024) 12:234. doi: 10.3390/microorganisms12020234, PMID: 38399637 PMC10891645

[24] Oelschlaeger TA. Mechanisms of probiotic actions–a review. Int J Med Microbiol. (2010) 300:57–62. doi: 10.1016/j.ijmm.2009.08.005 19783474

[25] Valdemiro Carlos S. The importance of prebiotics in functional foods and clinical practice. Food Nutr Sci. (2011) 2:33–144. doi: 10.4236/fns.2011.22019

[26] Dicksved J Schreiber O Willing B Petersson J Rang S Phillipson M. *Lactobacillus reuteri* maintains a functional mucosal barrier during DSS treatment despite mucus layer dysfunction. PLoS One. (2012) 7:e46399. doi: 10.1371/journal.pone.0046399, PMID: 23029509 PMC3459901

[27] Aghamohammad S Sepehr A Miri ST Najafi S Rohani M Pourshafiea MR. The effects of the probiotic cocktail on modulation of the NF-κB and JAK/STAT signaling pathways involved in the inflammatory response in bowel disease model. BMC Immunol. (2022) 23:1–10. doi: 10.1186/s12865-022-00484-6 35240996 PMC8896082

[28] Hrdý J Couturier-Maillard A Boutillier D Lapadatescu C Blanc P Procházka J. Oral supplementation with selected *Lactobacillus acidophilus* triggers IL-17-dependent innate defense response, activation of innate lymphoid cells type 3 and improves colitis. Sci Rep. (2022) 12:17591. doi: 10.1038/s41598-022-21643-0, PMID: 36266398 PMC9585059

[29] Qu S Fan L Qi Y Xu C Hu Y Chen S. *Akkermansia muciniphila* alleviates dextran sulfate sodium (DSS)-induced acute colitis by NLRP3 activation. Microbiol Spectr. (2021) 9:e00730–21. doi: 10.1128/Spectrum.00730-21 34612661 PMC8510245

[30] Yu P Ke C Guo J Zhang X Li B. *Lactobacillus plantarum* L15 alleviates colitis by inhibiting LPS-mediated NF-κB activation and ameliorates DSS-induced gut microbiota dysbiosis. Front Immunol. (2020) 11:575173. doi: 10.3389/fimmu.2020.575173, PMID: 33123156 PMC7566170

[31] Zhou X Liu H Zhang J Mu J Zalan Z Hegyi F. Protective effect of *Lactobacillus fermentum* CQPC04 on dextran sulfate sodium–induced colitis in mice is associated with modulation of the nuclear factor-κB signaling pathway. J Dairy Sci. (2019) 102:9570–85. doi: 10.3168/jds.2019-16840, PMID: 31477303

[32] Lee M Chang EB. Inflammatory bowel diseases (IBD) and the microbiome—searching the crime scene for clues. Gastroenterology. (2021) 160:524–37. doi: 10.1053/j.gastro.2020.09.056, PMID: 33253681 PMC8098834

[33] Tlaskalová-Hogenová H Tucková L Stepánková R Hudcovic T Palová-Jelínková L Kozáková H. Involvement of innate immunity in the development of inflammatory and autoimmune diseases. Ann N Y Acad Sci. (2005) 1051:787–98. doi: 10.1196/annals.1361.122 16127016

[34] Sartor RB. Therapeutic manipulation of the enteric microflora in inflammatory bowel diseases: antibiotics, probiotics, and prebiotics. Gastroenterology. (2004) 126:1620–33. doi: 10.1053/j.gastro.2004.03.024, PMID: 15168372

[35] Eun CS Mishima Y Wohlgemuth S Liu B Bower M Carroll IM. Induction of bacterial antigen-specific colitis by a simplified human microbiota consortium in gnotobiotic interleukin-10−/− mice. Infect Immun. (2014) 82:2239–46. doi: 10.1128/IAI.01513-13, PMID: 24643531 PMC4019192

[36] Guo XY Liu XJ Hao JY. Gut microbiota in ulcerative colitis: insights on pathogenesis and treatment. J Dig Dis. (2020) 21:147–59. doi: 10.1111/1751-2980.12849 32040250

[37] Clooney AG Eckenberger J Laserna-Mendieta E Sexton KA Bernstein MT Vagianos K. Ranking microbiome variance in inflammatory bowel disease: a large longitudinal intercontinental study. Gut. (2021) 70:499–510. doi: 10.1136/gutjnl-2020-321106 32536605 PMC7873428

[38] Goethel A Croitoru K Philpott DJ. The interplay between microbes and the immune response in inflammatory bowel disease. J Physiol. (2018) 596:3869–82. doi: 10.1113/JP275396, PMID: 29806140 PMC6117586

[39] Gibson GR Hutkins R Sanders ME Prescott SL Reimer RA Salminen SJ. Expert consensus document: the international scientific Association for Probiotics and Prebiotics (ISAPP) consensus statement on the definition and scope of prebiotics. Nat Rev Gastroenterol Hepatol. (2017) 14:491–502. doi: 10.1038/nrgastro.2017.75, PMID: 28611480

[40] Hill C Guarner F Reid G Gibson GR Merenstein DJ Pot B. Expert consensus document. The international scientific Association for Probiotics and Prebiotics consensus statement on the scope and appropriate use of the term probiotic. Nat Rev Gastroenterol Hepatol. (2014) 11:506–14. doi: 10.1038/nrgastro.2014.66, PMID: 24912386

[41] Hotel ACP Cordoba A. Health and nutritional properties of probiotics in food including powder milk with live lactic acid bacteria. Prevention. (2001) 5:1–10.

[42] Swanson KS Gibson GR Hutkins R Reimer RA Reid G Verbeke K. The international scientific Association for Probiotics and Prebiotics (ISAPP) consensus statement on the definition and scope of synbiotics. Nat Rev Gastroenterol Hepatol. (2020) 17:687–701. doi: 10.1038/s41575-020-0344-2, PMID: 32826966 PMC7581511

[43] Thomas CM Versalovic J. Probiotics-host communication: modulation of signaling pathways in the intestine. Gut Microbes. (2010) 1:148–63. doi: 10.4161/gmic.1.3.11712, PMID: 20672012 PMC2909492

[44] O'shea EF Cotter PD Stanton C Ross RP Hill C. Production of bioactive substances by intestinal bacteria as a basis for explaining probiotic mechanisms: bacteriocins and conjugated linoleic acid. Int J Food Microbiol. (2012) 152:189–205. doi: 10.1016/j.ijfoodmicro.2011.05.025, PMID: 21742394

[45] Spinler JK Taweechotipatr M Rognerud CL Ou CN Tumwasorn S Versalovic J. Human-derived probiotic *Lactobacillus reuteri* demonstrate antimicrobial activities targeting diverse enteric bacterial pathogens. Anaerobe. (2008) 14:166–71. doi: 10.1016/j.anaerobe.2008.02.001, PMID: 18396068 PMC2494590

[46] Lee BJ Bak Y-T. Irritable bowel syndrome, gut microbiota and probiotics. J Neurogastroenterol Motil. (2011) 17:252–66. doi: 10.5056/jnm.2011.17.3.252, PMID: 21860817 PMC3155061

[47] Bron PA Van Baarlen P Kleerebezem M. Emerging molecular insights into the interaction between probiotics and the host intestinal mucosa. Nat Rev Microbiol. (2012) 10:66–78. doi: 10.1038/nrmicro2690, PMID: 22101918

[48] Hemarajata P Versalovic J. Effects of probiotics on gut microbiota: mechanisms of intestinal immunomodulation and neuromodulation. Ther Adv Gastroenterol. (2013) 6:39–51. doi: 10.1177/1756283X12459294 PMC3539293 23320049

[49] Le B Yang SH. Efficacy of *Lactobacillus plantarum* in prevention of inflammatory bowel disease. Toxicol Rep. (2018) 5:314–7. doi: 10.1016/j.toxrep.2018.02.007, PMID: 29854599 PMC5977373

[50] Cheng F-S Pan D Chang B Jiang M Sang L-X. Probiotic mixture VSL# 3: An overview of basic and clinical studies in chronic diseases. World J Clin Cases. (2020) 8:1361–84. doi: 10.12998/wjcc.v8.i8.1361, PMID: 32368530 PMC7190945

[51] Diling C Xin Y Chaoqun Z Jian Y Xiaocui T Jun C. Extracts from *Hericium erinaceus* relieve inflammatory bowel disease by regulating immunity and gut microbiota. Oncotarget. (2017) 8:85838–57. doi: 10.18632/oncotarget.20689, PMID: 29156761 PMC5689651

[52] Gravina AG Pellegrino R Auletta S Palladino G Brandimarte G D’onofrio R. *Hericium erinaceus*, a medicinal fungus with a centuries-old history: evidence in gastrointestinal diseases. World J Gastroenterol. (2023) 29:3048–65. doi: 10.3748/wjg.v29.i20.3048, PMID: 37346156 PMC10280799

[53] Gravina AG Pellegrino R Palladino G Coppola A Brandimarte G Tuccillo C. *Hericium erinaceus*, in combination with natural flavonoid/alkaloid and B3/B8 vitamins, can improve inflammatory burden in inflammatory bowel diseases tissue: an *ex vivo* study. Front Immunol. (2023) 14:1215329. doi: 10.3389/fimmu.2023.1215329, PMID: 37465689 PMC10350490

[54] Qin M Geng Y Lu Z Xu H-Y Shi J-S Xu X. Anti-inflammatory effects of ethanol extract of Lion’s mane medicinal mushroom, *Hericium erinaceus* (Agaricomycetes), in mice with ulcerative colitis. Int J Med Mushrooms. (2016) 18:227–34. doi: 10.1615/IntJMedMushrooms.v18.i3.50 27481156

[55] Ren Z Xu Z Amakye WK Liu W Zhao Z Gao L. *Hericium erinaceus* mycelium-derived polysaccharide alleviates ulcerative colitis and modulates gut microbiota in Cynomolgus monkeys. Mol Nutr Food Res. (2023) 67:e2200450. doi: 10.1002/mnfr.202200450, PMID: 36443636

[56] Khan MA Tania M Liu R Rahman MM. *Hericium erinaceus*: an edible mushroom with medicinal values. J Complement Integr Med. (2013) 10:253–8. doi: 10.1515/jcim-2013-0001 23735479

[57] Chen L Zou Y Peng J Lu F Yin Y Li F. *Lactobacillus acidophilus* suppresses colitis-associated activation of the IL-23/Th17 axis. J Immunol Res. (2015) 2015:909514. doi: 10.1155/2015/909514 25973440 PMC4417982

[58] Chen Y Cui W Li X Yang H. Interaction between commensal bacteria, immune response and the intestinal barrier in inflammatory bowel disease. Front Immunol. (2021) 12:761981. doi: 10.3389/fimmu.2021.761981 34858414 PMC8632219

[59] Qin W Luo H Yang L Hu D Jiang S-P Peng D-Y. Rubia cordifolia L. ameliorates DSS-induced ulcerative colitis in mice through dual inhibition of NLRP3 inflammasome and IL-6/JAK2/STAT3 pathways. Heliyon. (2022) 8:e10314. doi: 10.1016/j.heliyon.2022.e10314, PMID: 36082330 PMC9445285

[60] Wu Y Jha R Li A Liu H Zhang Z Zhang C. Probiotics (*Lactobacillus plantarum* HNU082) supplementation relieves ulcerative colitis by affecting intestinal barrier functions, immunity-related gene expression, gut microbiota, and metabolic pathways in mice. Microbiol Spectr. (2022) 10:e01651–22. doi: 10.1128/spectrum.01651-22 36321893 PMC9769980

[61] Giri R Hoedt EC Khushi S Salim AA Bergot A-S Schreiber V. Secreted NF-κB suppressive microbial metabolites modulate gut inflammation. Cell Rep. (2022) 39:110646. doi: 10.1016/j.celrep.2022.110646, PMID: 35417687

[62] Chen Z Yi L Pan Y Long X Mu J Yi R. *Lactobacillus fermentum* ZS40 ameliorates inflammation in mice with ulcerative colitis induced by dextran sulfate sodium. Front Pharmacol. (2021) 12:700217. doi: 10.3389/fphar.2021.700217, PMID: 34867317 PMC8640127

[63] Sun M Liu Y Song Y Gao Y Zhao F Luo Y. The ameliorative effect of *Lactobacillus plantarum*-12 on DSS-induced murine colitis. Food Funct. (2020) 11:5205–22. doi: 10.1039/D0FO00007H, PMID: 32458908

[64] Dai C Zheng C-Q Meng F-J Zhou Z Sang L-X Jiang M. VSL# 3 probiotics exerts the anti-inflammatory activity *via* PI3k/Akt and NF-κB pathway in rat model of DSS-induced colitis. Mol Cell Biochem. (2013) 374:1–11. doi: 10.1007/s11010-012-1488-3 23271629

[65] Dong L Wang M Guo J Wang J. Influences of probiotics combined with sulfasalazine on rats with ulcerative colitis *via* the Wnt/β-catenin signaling pathway. Eur Rev Med Pharmacol Sci. (2019) 23:6371–8. doi: 10.26355/eurrev_201907_18461, PMID: 31364145

[66] Damoogh S Vosough M Hadifar S Rasoli M Gorjipour A Falsafi S. Evaluation of *E. coli* Nissle1917 derived metabolites in modulating key mediator genes of the TLR signaling pathway. BMC Res Notes. (2021) 14:156. doi: 10.1186/s13104-021-05568-x, PMID: 33902702 PMC8077910

[67] Leccese G Bibi A Mazza S Facciotti F Caprioli F Landini P. Probiotic Lactobacillus and Bifidobacterium strains counteract adherent-invasive *Escherichia coli* (AIEC) virulence and hamper IL-23/Th17 axis in ulcerative colitis, but not in crohn’s disease. Cells. (2020) 9:1824. doi: 10.3390/cells9081824, PMID: 32752244 PMC7464949

[68] Li Y Yang S Lun J Gao J Gao X Gong Z. Inhibitory effects of the *Lactobacillus rhamnosus* GG effector protein HM0539 on inflammatory response through the TLR4/MyD88/NF-кB axis. Front Immunol. (2020) 11:551449. doi: 10.3389/fimmu.2020.551449, PMID: PMC7573360 33123130

[69] Kanmani P Kim H. Beneficial effect of immunobiotic strains on attenuation of Salmonella induced inflammatory response in human intestinal epithelial cells. PLoS One. (2020) 15:e0229647. doi: 10.1371/journal.pone.0229647, PMID: 32150574 PMC7062243

[70] Liu T Zhang L Joo D Sun S-C. NF-κB signaling in inflammation. Signal Transduct Target Ther. (2017) 2:1–9. doi: 10.1038/sigtrans.2017.23 PMC5661633 29158945

[71] Yu H Lin L Zhang Z Zhang H Hu H. Targeting NF-κB pathway for the therapy of diseases: mechanism and clinical study. Signal Transduct Target Ther. (2020) 5:209. doi: 10.1038/s41392-020-00312-6, PMID: 32958760 PMC7506548

[72] Sun S-C. Non-canonical NF-κB signaling pathway. Cell Res. (2011) 21:71–85. doi: 10.1038/cr.2010.177 21173796 PMC3193406

[73] Vallabhapurapu S Karin M. Regulation and function of NF-κB transcription factors in the immune system. Annu Rev Immunol. (2009) 27:693–733. doi: 10.1146/annurev.immunol.021908.132641 19302050

[74] Zhang H Sun S-C. NF-κB in inflammation and renal diseases. Cell Biosci. (2015) 5:1–12. doi: 10.1186/s13578-015-0056-4 26579219 PMC4647710

[75] Sun S-C Ley SC. New insights into NF-κB regulation and function. Trends Immunol. (2008) 29:469–78. doi: 10.1016/j.it.2008.07.003, PMID: 18775672 PMC5751948

[76] Israël A. The IKK complex, a central regulator of NF-κB activation. Cold Spring Harb Perspect Biol. (2010) 2:a000158. doi: 10.1101/cshperspect.a000158 20300203 PMC2829958

[77] Hayden MS Ghosh S. Shared principles in NF-κB signaling. Cell. (2008) 132:344–62. doi: 10.1016/j.cell.2008.01.020 18267068

[78] Sc BSL. Functions of NF-kappaB 1 and NF-kappaB2 in immune cell biology. Bioehem J. (2004) 382:393–409. doi: 10.1042/BJ20040544 PMC1133795 15214841

[79] Senftleben U Cao Y Xiao G Greten FR KräHn G Bonizzi G. Activation by IKKα of a second, evolutionary conserved, NF-κB signaling pathway. Science. (2001) 293:1495–9. doi: 10.1126/science.1062677, PMID: 11520989

[80] Xiao G Harhaj EW Sun S-C. NF-κB-inducing kinase regulates the processing of NF-κB2 p100. Mol Cell. (2001) 7:401–9. doi: 10.1016/S1097-2765(01)00187-3 11239468

[81] Cildir G Low KC Tergaonkar V. Noncanonical NF-κB signaling in health and disease. Trends Mol Med. (2016) 22:414–29. doi: 10.1016/j.molmed.2016.03.002, PMID: 27068135

[82] Sun S-C Liu Z-G. A special issue on NF-κB signaling and function. Cell Res. (2011) 21:1–2. doi: 10.1038/cr.2011.1, PMID: 21196938 PMC3193411

[83] Hegazy SK El-Bedewy MM. Effect of probiotics on pro-inflammatory cytokines and NF-κB activation in ulcerative colitis. World J Gastroenterol: WJG. (2010) 16:4145–51. doi: 10.3748/wjg.v16.i33.4145, PMID: 20806430 PMC2932917

[84] Sakthivel KM Guruvayoorappan C. Protective effect of Acacia ferruginea against ulcerative colitis *via* modulating inflammatory mediators, cytokine profile and NF-κB signal transduction pathways. J Environ Pathol Toxicol Oncol. (2014) 33:83–98. doi: 10.1615/JEnvironPatholToxicolOncol.2014008425, PMID: 24941292

[85] Wang X Liu Y Dong H Wu L Feng X Zhou Z. Herb-partitioned moxibustion regulates the TLR2/NF-κB signaling pathway in a rat model of ulcerative colitis. Evid Based Complement Alternat Med. (2015) 2015:949065. doi: 10.1155/2015/949065 26339273 PMC4538972

[86] Yu Z-H Huang F Xu N Zhao D-M Hu F-A Liu J. Expression of toll-like receptor 4, CD14, and NF-κB in Chinese patients with ulcerative colitis. J Immunoass Immunochem. (2011) 32:47–56. doi: 10.1080/15321819.2010.538108, PMID: 21253969

[87] Li Z Zhang DK Yi WQ Ouyang Q Chen YQ Gan HT. NF-κB p65 antisense oligonucleotides may serve as a novel molecular approach for the treatment of patients with ulcerative colitis. Arch Med Res. (2008) 39:729–34. doi: 10.1016/j.arcmed.2008.08.001 18996285

[88] Vereecke L Vieira-Silva S Billiet T Van Es JH Mc Guire C Slowicka K. A20 controls intestinal homeostasis through cell-specific activities. Nat Commun. (2014) 5:5103. doi: 10.1038/ncomms6103 25267258

[89] Zhang J Stirling B Temmerman ST Ma CA Fuss IJ Derry JM. Impaired regulation of NF-κB and increased susceptibility to colitis-associated tumorigenesis in CYLD-deficient mice. J Clin Invest. (2006) 116:3042–9. doi: 10.1172/JCI28746, PMID: 17053834 PMC1616194

[90] Fichtner-Feigl S Fuss IJ Preiss JC Strober W Kitani A. Treatment of murine Th1-and Th2-mediated inflammatory bowel disease with NF-κB decoy oligonucleotides. J Clin Invest. (2005) 115:3057–71. doi: 10.1172/JCI24792, PMID: 16239967 PMC1257534

[91] Neurath MF Pettersson S Meyer Zum Büschenfelde K-H Strober W. Local administration of antisense phosphorothiate olignucleotides to the p65 subunit of NF–κB abrogates established experimental colitis in mice. Nat Med. (1996) 2:998–1004. doi: 10.1038/nm0996-998, PMID: 8782457

[92] Greten FR Eckmann L Greten TF Park JM Li Z-W Egan LJ. IKKβ links inflammation and tumorigenesis in a mouse model of colitis-associated cancer. Cell. (2004) 118:285–96. doi: 10.1016/j.cell.2004.07.013, PMID: 15294155

[93] Nenci A Becker C Wullaert A Gareus R Van Loo G Danese S. Epithelial NEMO links innate immunity to chronic intestinal inflammation. Nature. (2007) 446:557–61. doi: 10.1038/nature05698, PMID: 17361131

[94] Zaph C Troy AE Taylor BC Berman-Booty LD Guild KJ Du Y. Epithelial-cell-intrinsic IKK-β expression regulates intestinal immune homeostasis. Nature. (2007) 446:552–6. doi: 10.1038/nature05590, PMID: 17322906

[95] Vlantis K Wullaert A Polykratis A Kondylis V Dannappel M Schwarzer R. NEMO prevents RIP kinase 1-mediated epithelial cell death and chronic intestinal inflammation by NF-κB-dependent and-independent functions. Immunity. (2016) 44:553–67. doi: 10.1016/j.immuni.2016.02.020 26982364 PMC4803910

[96] Ali FE Ibrahim IM Ghogar OM Abd-Alhameed EK Althagafy HS Hassanein EH. Therapeutic interventions target the NLRP3 inflammasome in ulcerative colitis: comprehensive study. World J Gastroenterol. (2023) 29:1026–53. doi: 10.3748/wjg.v29.i6.1026, PMID: 36844140 PMC9950862

[97] Gong T Liu L Jiang W Zhou R. DAMP-sensing receptors in sterile inflammation and inflammatory diseases. Nat Rev Immunol. (2020) 20:95–112. doi: 10.1038/s41577-019-0215-7 31558839

[98] Guo H Callaway JB Ting JP. Inflammasomes: mechanism of action, role in disease, and therapeutics. Nat Med. (2015) 21:677–87. doi: 10.1038/nm.3893, PMID: 26121197 PMC4519035

[99] Menu P Vince J. The NLRP3 inflammasome in health and disease: the good, the bad and the ugly. Clin Exp Immunol. (2011) 166:1–15. doi: 10.1111/j.1365-2249.2011.04440.x, PMID: 21762124 PMC3193914

[100] Rheinheimer J De Souza BM Cardoso NS Bauer AC Crispim D. Current role of the NLRP3 inflammasome on obesity and insulin resistance: a systematic review. Metabolism. (2017) 74:1–9. doi: 10.1016/j.metabol.2017.06.002 28764843

[101] Strowig T Henao-Mejia J Elinav E Flavell R. Inflammasomes in health and disease. Nature. (2012) 481:278–86. doi: 10.1038/nature10759, PMID: 22258606

[102] Sun H-J Ren X-S Xiong X-Q Chen Y-Z Zhao M-X Wang J-J. NLRP3 inflammasome activation contributes to VSMC phenotypic transformation and proliferation in hypertension. Cell Death Dis. (2017) 8:e3074–4. doi: 10.1038/cddis.2017.470, PMID: 28981106 PMC5680591

[103] De Nardo D Latz E. NLRP3 inflammasomes link inflammation and metabolic disease. Trends Immunol. (2011) 32:373–9. doi: 10.1016/j.it.2011.05.004, PMID: 21733753 PMC3151541

[104] Ali F Abo-Youssef A Messiha B Hemeda R. Protective effects of quercetin and ursodeoxycholic acid on hepatic ischemiareperfusion injury in rats. Clin Pharmacol Biopharm. (2015) 4. doi: 10.4172/2167-065X.1000128

[105] Hanaei S Sadr M Rezaei A Shahkarami S Daryani NE Bidoki A. Association of NLRP3 single nucleotide polymorphisms with ulcerative colitis: a case-control study. Clin Res Hepatol Gastroenterol. (2018) 42:269–75. doi: 10.1016/j.clinre.2017.09.003, PMID: 29102545

[106] Yoganathan P Rossel J-B Jordi SBU Franc Y Biedermann L Misselwitz B. Genotype–phenotype associations of polymorphisms within the gene locus of NOD-like receptor pyrin domain containing 3 in Swiss inflammatory bowel disease patients. BMC Gastroenterol. (2021) 21:310. doi: 10.1186/s12876-021-01880-9, PMID: 34344313 PMC8336111

[107] Ghoreschi K Laurence A O’shea JJ. Janus kinases in immune cell signaling. Immunol Rev. (2009) 228:273–87. doi: 10.1111/j.1600-065X.2008.00754.x, PMID: 19290934 PMC2782696

[108] Kiu H Nicholson SE. Biology and significance of the JAK/STAT signalling pathways. Growth Factors. (2012) 30:88–106. doi: 10.3109/08977194.2012.660936, PMID: 22339650 PMC3762697

[109] Caiazzo G Caiazzo A Napolitano M Megna M Potestio L Fornaro L. The use of JAK/STAT inhibitors in chronic inflammatory disorders. J Clin Med. (2023) 12:2865. doi: 10.3390/jcm12082865, PMID: 37109202 PMC10142234

[110] Ihle JN. The stat family in cytokine signaling. Curr Opin Cell Biol. (2001) 13:211–7. doi: 10.1016/S0955-0674(00)00199-X 11248555

[111] Yu H Pardoll D Jove R. STATs in cancer inflammation and immunity: a leading role for STAT3. Nat Rev Cancer. (2009) 9:798–809. doi: 10.1038/nrc2734, PMID: 19851315 PMC4856025

[112] Duetsch G Illig T Loesgen S Rohde K Klopp N Herbon N. STAT6 as an asthma candidate gene: polymorphism-screening, association and haplotype analysis in a Caucasian sib-pair study. Hum Mol Genet. (2002) 11:613–21. doi: 10.1093/hmg/11.6.613, PMID: 11912176

[113] Miyagi T Gil MP Wang X Louten J Chu W-M Biron CA. High basal STAT4 balanced by STAT1 induction to control type 1 interferon effects in natural killer cells. J Exp Med. (2007) 204:2383–96. doi: 10.1084/jem.20070401, PMID: 17846149 PMC2118450

[114] Neubauer H Cumano A Müller M Wu H Huffstadt U Pfeffer K. Jak2 deficiency defines an essentialdevelopmental checkpoint in definitivehematopoiesis. Cell. (1998) 93:397–409. doi: 10.1016/S0092-8674(00)81168-X, PMID: 9590174

[115] Rodig SJ Meraz MA White JM Lampe PA Riley JK Arthur CD. Disruption of the Jak1 gene demonstrates obligatory and nonredundant roles of the Jaks in cytokine-induced biologic responses. Cell. (1998) 93:373–83. doi: 10.1016/S0092-8674(00)81166-6, PMID: 9590172

[116] Karaghiosoff M Neubauer H Lassnig C Kovarik P Schindler H Pircher H. Partial impairment of cytokine responses in Tyk2-deficient mice. Immunity. (2000) 13:549–60. doi: 10.1016/S1074-7613(00)00054-6, PMID: 11070173

[117] Masuda A Matsuguchi T Yamaki K Hayakawa T Kubo M Larochelle WJ. Interleukin-15 induces rapid tyrosine phosphorylation of STAT6 and the expression of interleukin-4 in mouse mast cells. J Biol Chem. (2000) 275:29331–7. doi: 10.1074/jbc.M910290199, PMID: 10882748

[118] Udy GB Towers RP Snell RG Wilkins RJ Park S-H Ram PA. Requirement of STAT5b for sexual dimorphism of body growth rates and liver gene expression. Proc Natl Acad Sci. (1997) 94:7239–44. doi: 10.1073/pnas.94.14.7239, PMID: 9207075 PMC23803

[119] Tanaka Y Luo Y O’shea JJ Nakayamada S. Janus kinase-targeting therapies in rheumatology: a mechanisms-based approach. Nat Rev Rheumatol. (2022) 18:133–45. doi: 10.1038/s41584-021-00726-8, PMID: 34987201 PMC8730299

[120] Nemeth ZH Bogdanovski DA Barratt-Stopper P Paglinco SR Antonioli L Rolandelli RH. Crohn’s disease and ulcerative colitis show unique cytokine profiles. Cureus. (2017) 9:e1177. doi: 10.7759/cureus.1177 28533995 PMC5438231

[121] Salas A Hernandez-Rocha C Duijvestein M Faubion W Mcgovern D Vermeire S. JAK–STAT pathway targeting for the treatment of inflammatory bowel disease. Nat Rev Gastroenterol Hepatol. (2020) 17:323–37. doi: 10.1038/s41575-020-0273-0 32203403

[122] O'sullivan LA Liongue C Lewis RS Stephenson SE Ward AC. Cytokine receptor signaling through the Jak–stat–Socs pathway in disease. Mol Immunol. (2007) 44:2497–506. doi: 10.1016/j.molimm.2006.11.025, PMID: 17208301

[123] Coulombe P Meloche S. Atypical mitogen-activated protein kinases: structure, regulation and functions. Biochim Biophys Acta. (2007) 1773:1376–87. doi: 10.1016/j.bbamcr.2006.11.001 17161475

[124] Sun Y Liu W-Z Liu T Feng X Yang N Zhou H-F. Signaling pathway of MAPK/ERK in cell proliferation, differentiation, migration, senescence and apoptosis. J Recept Signal Transduct Res. (2015) 35:600–4. doi: 10.3109/10799893.2015.1030412, PMID: 26096166

[125] Liu F Yang X Geng M Huang M. Targeting ERK, an Achilles' heel of the MAPK pathway, in cancer therapy. Acta Pharm Sin B. (2018) 8:552–62. doi: 10.1016/j.apsb.2018.01.008, PMID: 30109180 PMC6089851

[126] Sui X Kong N Ye L Han W Zhou J Zhang Q. p38 and JNK MAPK pathways control the balance of apoptosis and autophagy in response to chemotherapeutic agents. Cancer Lett. (2014) 344:174–9. doi: 10.1016/j.canlet.2013.11.019, PMID: 24333738

[127] Docena G Rovedatti L Kruidenier L Fanning A Leakey N Knowles C. Down-regulation of p38 mitogen-activated protein kinase activation and proinflammatory cytokine production by mitogen-activated protein kinase inhibitors in inflammatory bowel disease. Clin Exp Immunol. (2010) 162:108–15. doi: 10.1111/j.1365-2249.2010.04203.x, PMID: 20731675 PMC2990936

[128] Kaminska B Gozdz A Zawadzka M Ellert-Miklaszewska A Lipko M. MAPK signal transduction underlying brain inflammation and gliosis as therapeutic target. Anat Rec. (2009) 292:1902–13. doi: 10.1002/ar.21047, PMID: 19943344

[129] Moens U Kostenko S Sveinbjørnsson B. The role of mitogen-activated protein kinase-activated protein kinases (MAPKAPKs) in inflammation. Genes. (2013) 4:101–33. doi: 10.3390/genes4020101, PMID: 24705157 PMC3899974

[130] Moparthi L Koch S. Wnt signaling in intestinal inflammation. Differentiation. (2019) 108:24–32. doi: 10.1016/j.diff.2019.01.002 30718056

[131] You J Nguyen AV Albers CG Lin F Holcombe RF. Wnt pathway-related gene expression in inflammatory bowel disease. Dig Dis Sci. (2008) 53:1013–9. doi: 10.1007/s10620-007-9973-3, PMID: 17939044

[132] Hughes K Sablitzky F Mahida Y. Expression profiling of Wnt family of genes in normal and inflammatory bowel disease primary human intestinal myofibroblasts and normal human colonic crypt epithelial cells. Inflamm Bowel Dis. (2011) 17:213–20. doi: 10.1002/ibd.21353, PMID: 20848536

[133] Serafino A Moroni N Zonfrillo M Andreola F Mercuri L Nicotera G. WNT-pathway components as predictive markers useful for diagnosis, prevention and therapy in inflammatory bowel disease and sporadic colorectal cancer. Oncotarget. (2014) 5:978–92. doi: 10.18632/oncotarget.1571, PMID: 24657851 PMC4011599

[134] Bradford EM Ryu SH Singh AP Lee G Goretsky T Sinh P. Epithelial TNF receptor signaling promotes mucosal repair in inflammatory bowel disease. J Immunol. (2017) 199:1886–97. doi: 10.4049/jimmunol.1601066, PMID: 28747340 PMC5568528

[135] Gregorieff A Pinto D Begthel H Destrée O Kielman M Clevers H. Expression pattern of Wnt signaling components in the adult intestine. Gastroenterology. (2005) 129:626–38. doi: 10.1016/j.gastro.2005.06.007, PMID: 16083717

[136] Greicius G Virshup DM. Stromal control of intestinal development and the stem cell niche. Differentiation. (2019) 108:8–16. doi: 10.1016/j.diff.2019.01.001, PMID: 30683451

[137] Koch S. Extrinsic control of Wnt signaling in the intestine. Differentiation. (2017) 97:1–8. doi: 10.1016/j.diff.2017.08.003, PMID: 28802143

[138] Noviello D Mager R Roda G Borroni RG Fiorino G Vetrano S. The IL23-IL17 immune axis in the treatment of ulcerative colitis: successes, defeats, and ongoing challenges. Front Immunol. (2021) 12:1735. doi: 10.3389/fimmu.2021.611256 PMC8165319 34079536

[139] Arnold IC Mathisen S Schulthess J Danne C Hegazy AN Powrie F. CD11c+ monocyte/macrophages promote chronic *Helicobacter hepaticus*-induced intestinal inflammation through the production of IL-23. Mucosal Immunol. (2016) 9:352–63. doi: 10.1038/mi.2015.65, PMID: 26242598 PMC4650208

[140] Karaboga İ Demirtas S Karaca T. Investigation of the relationship between the Th17/IL-23 pathway and innate-adaptive immune system in TNBS-induced colitis in rats. Iran J Basic Med Sci. (2017) 20:870. doi: 10.22038/IJBMS.2017.9108 29085578 PMC5651472

[141] Yen D Cheung J Scheerens H Poulet F Mcclanahan T Mckenzie B. IL-23 is essential for T cell–mediated colitis and promotes inflammation *via* IL-17 and IL-6. J Clin Invest. (2006) 116:1310–6. doi: 10.1172/JCI21404, PMID: 16670770 PMC1451201

[142] Leppkes M Becker C Ivanov II Hirth S Wirtz S Neufert C. RORγ-expressing Th17 cells induce murine chronic intestinal inflammation *via* redundant effects of IL-17A and IL-17F. Gastroenterology. (2009) 136:257–67. doi: 10.1053/j.gastro.2008.10.018, PMID: 18992745

[143] Yang XO Chang SH Park H Nurieva R Shah B Acero L. Regulation of inflammatory responses by IL-17F. J Exp Med. (2008) 205:1063–75. doi: 10.1084/jem.20071978, PMID: 18411338 PMC2373839

[144] Zhang Z Zheng M Bindas J Schwarzenberger P Kolls JK. Critical role of IL-17 receptor signaling in acute TNBS-induced colitis. Inflamm Bowel Dis. (2006) 12:382–8. doi: 10.1097/01.MIB.0000218764.06959.91, PMID: 16670527

[145] Gheita TA El Gazzar II El-Fishawy HS Aboul-Ezz MA Kenawy SA. Involvement of IL-23 in enteropathic arthritis patients with inflammatory bowel disease: preliminary results. Clin Rheumatol. (2014) 33:713–7. doi: 10.1007/s10067-013-2469-y, PMID: 24384828

[146] Öhman L Dahlén R Isaksson S Sjöling Å Wick M-J Sjövall H. Serum IL-17A in newly diagnosed treatment-naive patients with ulcerative colitis reflects clinical disease severity and predicts the course of disease. Inflamm Bowel Dis. (2013) 19:2433–9. doi: 10.1097/MIB.0b013e3182a563cb, PMID: 23966065

[147] Rafa H Saoula H Belkhelfa M Medjeber O Soufli I Toumi R. IL-23/IL-17A axis correlates with the nitric oxide pathway in inflammatory bowel disease: immunomodulatory effect of retinoic acid. J Interf Cytokine Res. (2013) 33:355–68. doi: 10.1089/jir.2012.0063, PMID: 23472658

[148] Youssef T Saleh SA Rund A Montasser I Mohsen M Hazem O. Evaluation of interleukin 23 (IL-23) as a non-invasive test of disease severity in patients with ulcerative colitis. Arab J Gastroenterol. (2018) 19:116–20. doi: 10.1016/j.ajg.2018.09.003, PMID: 30268427

[149] Zheng Z Wan X Liu L. Serum contents of IL-23 and IL-17 in the patients with ulcerative colitis and the clinical significance. Chin J Cell Mol Immunol. (2011) 27:203–6. PMID: 21560441

[150] Zhu X-M Shi Y-Z Cheng M Wang D-F Fan J-F. Serum IL-6, IL-23 profile and Treg/Th17 peripheral cell populations in pediatric patients with inflammatory bowel disease. Pharmazie. (2017) 72:283–7. doi: 10.1691/ph.2017.6957 29441874

[151] Sugihara T Kobori A Imaeda H Tsujikawa T Amagase K Takeuchi K. The increased mucosal mRNA expressions of complement C3 and interleukin-17 in inflammatory bowel disease. Clin Exp Immunol. (2010) 160:386–93. doi: 10.1111/j.1365-2249.2010.04093.x, PMID: 20089077 PMC2883109

[152] Neurath MF Fuss I Kelsall BL Stüber E Strober W. Antibodies to interleukin 12 abrogate established experimental colitis in mice. J Exp Med. (1995) 182:1281–90. doi: 10.1084/jem.182.5.1281, PMID: 7595199 PMC2192205

[153] Noguchi D Wakita D Tajima M Ashino S Iwakura Y Zhang Y. Blocking of IL-6 signaling pathway prevents CD4+ T cell-mediated colitis in a Th17-independent manner. Int Immunol. (2007) 19:1431–40. doi: 10.1093/intimm/dxm114, PMID: 17981790

[154] Shi X-Z Lindholm PF Sarna SK. NF-κB activation by oxidative stress and inflammation suppresses contractility in colonic circular smooth muscle cells. Gastroenterology. (2003) 124:1369–80. doi: 10.1016/S0016-5085(03)00263-4, PMID: 12730877

[155] Kanda Y Osaki M Okada F. Chemopreventive strategies for inflammation-related carcinogenesis: current status and future direction. Int J Mol Sci. (2017) 18:867. doi: 10.3390/ijms18040867, PMID: 28422073 PMC5412448

[156] Lopetuso LR Chowdhry S Pizarro TT. Opposing functions of classic and novel IL-1 family members in gut health and disease. Front Immunol. (2013) 4:181. doi: 10.3389/fimmu.2013.00181 23847622 PMC3705591

[157] Hu X Han C Jin J Qin K Zhang H Li T. Integrin CD11b attenuates colitis by strengthening Src-Akt pathway to polarize anti-inflammatory IL-10 expression. Sci Rep. (2016) 6:1–11. doi: 10.1038/srep26252 27188220 PMC4870583

[158] Zong M Chang C Anjum R Xu H Guo Y Pan D. Multifunctional LPxTG-motif surface protein derived from *Limosilactobacillus reuteri* SH 23 in DSS-induced ulcerative colitis of mice. FASEB J. (2022) 36:e22421–1. doi: 10.1096/fj.202200252 37000564

[159] Gao R Shen Y Shu W Jin W Bai F Wang J. Sturgeon hydrolysates alleviate DSS-induced colon colitis in mice by modulating NF-κB, MAPK, and microbiota composition. Food Funct. (2020) 11:6987–99. doi: 10.1039/C9FO02772F, PMID: 32701080

[160] Shi R Yu F Hu X Liu Y Jin Y Ren H. Protective effect of *Lactiplantibacillus plantarum* subsp. plantarum SC-5 on dextran sulfate sodium—induced colitis in mice. Food Secur. (2023) 12:897. doi: 10.3390/foods12040897, PMID: 36832972 PMC9957050

[161] Su L Ma F An Z Ji X Zhang P Yue Q. The metabolites of *Lactobacillus fermentum* F-B9-1 relieved dextran sulfate sodium-induced experimental ulcerative colitis in mice. Front Microbiol. (2022) 13:865925. doi: 10.3389/fmicb.2022.865925, PMID: 35572623 PMC9096258

[162] Zhou X Münch G Wohlmuth H Afzal S Kao M-HT Al-Khazaleh A. Synergistic inhibition of pro-inflammatory pathways by ginger and turmeric extracts in RAW 264.7 cells. Front Pharmacol. (2022) 13:818166. doi: 10.3389/fphar.2022.818166 35662723 PMC9160922

[163] Chung Y-W Choi J-H Oh TY Eun C Han DS. *Lactobacillus casei* prevents the development of dextran sulphate sodium-induced colitis in toll-like receptor 4 mutant mice. Clin Exp Immunol. (2008) 151:182–9. doi: 10.1111/j.1365-2249.2007.03549.x, PMID: 18005362 PMC2276925

[164] Lange S Delbro DS Jennische E Mattsby-Baltzer I. The role of the Lps gene in experimental ulcerative colitis in mice. APMIS. (1996) 104:823–33. doi: 10.1111/j.1699-0463.1996.tb04948.x 8982246

[165] Rakoff-Nahoum S Paglino J Eslami-Varzaneh F Edberg S Medzhitov R. Recognition of commensal microflora by toll-like receptors is required for intestinal homeostasis. Cell. (2004) 118:229–41. doi: 10.1016/j.cell.2004.07.002, PMID: 15260992

[166] Araki A Kanai T Ishikura T Makita S Uraushihara K Iiyama R. MyD88-deficient mice develop severe intestinal inflammation in dextran sodium sulfate colitis. J Gastroenterol. (2005) 40:16–23. doi: 10.1007/s00535-004-1492-9, PMID: 15692785

[167] Alex P Zachos NC Nguyen T Gonzales L Chen T-E Conklin LS. Distinct cytokine patterns identified from multiplex profiles of murine DSS and TNBS-induced colitis. Inflamm Bowel Dis. (2009) 15:341–52. doi: 10.1002/ibd.20753, PMID: 18942757 PMC2643312

[168] Boussenna A Goncalves-Mendes N Joubert-Zakeyh J Pereira B Fraisse D Vasson M-P. Impact of basal diet on dextran sodium sulphate (DSS)-induced colitis in rats. Eur J Nutr. (2015) 54:1217–27. doi: 10.1007/s00394-014-0800-2 25410748

[169] Geier MS Butler RN Giffard PM Howarth GS. *Lactobacillus fermentum* BR11, a potential new probiotic, alleviates symptoms of colitis induced by dextran sulfate sodium (DSS) in rats. Int J Food Microbiol. (2007) 114:267–74. doi: 10.1016/j.ijfoodmicro.2006.09.018, PMID: 17150273

[170] Kumar CS Reddy KK Reddy AG Vinoth A Ch SRC Boobalan G. Protective effect of *Lactobacillus plantarum* 21, a probiotic on trinitrobenzenesulfonic acid-induced ulcerative colitis in rats. Int Immunopharmacol. (2015) 25:504–10. doi: 10.1016/j.intimp.2015.02.026, PMID: 25727887

[171] Wang N Wang H Yao H Wei Q Mao X-M Jiang T. Expression and activity of the TLR4/NF-κB signaling pathway in mouse intestine following administration of a short-term high-fat diet. Exp Ther Med. (2013) 6:635–40. doi: 10.3892/etm.2013.1214, PMID: 24137239 PMC3786917

[172] Wang Y Tu Q Yan W Xiao D Zeng Z Ouyang Y. CXC195 suppresses proliferation and inflammatory response in LPS-induced human hepatocellular carcinoma cells *via* regulating TLR4-MyD88-TAK1-mediated NF-κB and MAPK pathway. Biochem Biophys Res Commun. (2015) 456:373–9. doi: 10.1016/j.bbrc.2014.11.090, PMID: 25475726

[173] Makarov SS. NF-κB as a therapeutic target in chronic inflammation: recent advances. Mol Med Today. (2000) 6:441–8. doi: 10.1016/S1357-4310(00)01814-1 11074370

[174] Tak P Firestein G. NF-B: a key role in inflammatory diseases. J Clin Invest. (2001) 107:7–11. doi: 10.1172/JCI11830, PMID: 11134171 PMC198552

[175] Chen L Lin M-J Zhan L-L Lv X-P. Analysis of TLR4 and TLR2 polymorphisms in inflammatory bowel disease in a Guangxi Zhuang population. World J Gastroenterol: WJG. (2012) 18:6856–60. doi: 10.3748/wjg.v18.i46.6856, PMID: 23239925 PMC3520176

[176] Rovedatti L Kudo T Biancheri P Sarra M Knowles C Rampton DS. Differential regulation of interleukin 17 and interferon γ production in inflammatory bowel disease. Gut. (2009) 58:1629–36. doi: 10.1136/gut.2009.182170, PMID: 19740775

[177] Xia Y Chen Y Wang G Yang Y Song X Xiong Z. *Lactobacillus plantarum* AR113 alleviates DSS-induced colitis by regulating the TLR4/MyD88/NF-κB pathway and gut microbiota composition. J Funct Foods. (2020) 67:103854. doi: 10.1016/j.jff.2020.103854

[178] Liu G Yu L Fang J Hu C-AA Yin J Ni H. Methionine restriction on oxidative stress and immune response in dss-induced colitis mice. Oncotarget. (2017) 8:44511–20. doi: 10.18632/oncotarget.17812, PMID: 28562346 PMC5546498

[179] Laird MH Rhee SH Perkins DJ Medvedev AE Piao W Fenton MJ. TLR4/MyD88/PI3K interactions regulate TLR4 signaling. J Leukoc Biol. (2009) 85:966–77. doi: 10.1189/jlb.1208763, PMID: 19289601 PMC2698589

[180] Chung H-T Choi B-M Kwon Y-G Kim Y-M. Interactive relations between nitric oxide (NO) and carbon monoxide (CO): heme oxygenase-1/CO pathway is a key modulator in NO-mediated antiapoptosis and anti-inflammation. Methods Enzymol. (2008) 441:329–38. doi: 10.1016/S0076-6879(08)01218-4 18554543

[181] Mandal P Roychowdhury S Park P-H Pratt BT Roger T Nagy LE. Adiponectin and heme oxygenase-1 suppress TLR4/MyD88-independent signaling in rat Kupffer cells and in mice after chronic ethanol exposure. J Immunol. (2010) 185:4928–37. doi: 10.4049/jimmunol.1002060, PMID: 20861358 PMC5085268

[182] Wang N Wang S Xu B Liu F Huo G Li B. Alleviation effects of *Bifidobacterium animalis* subsp. lactis XLTG11 on dextran sulfate sodium-induced colitis in mice. Microorganisms. (2021) 9:2093. doi: 10.3390/microorganisms9102093 34683415 PMC8539219

[183] Aximujiang K Kaheman K Wushouer X Wu G Ahemaiti A Yunusi K. *Lactobacillus acidophilus* and HKL suspension alleviates ulcerative colitis in rats by regulating gut microbiota, suppressing TLR9, and promoting metabolism. Front Pharmacol. (2022) 13:859628. doi: 10.3389/fphar.2022.859628, PMID: 35600873 PMC9118348

[184] Pedersen G Andresen L Matthiessen M Rask-Madsen J Brynskov J. Expression of toll-like receptor 9 and response to bacterial CpG oligodeoxynucleotides in human intestinal epithelium. Clin Exp Immunol. (2005) 141:298–306. doi: 10.1111/j.1365-2249.2005.02848.x, PMID: 15996194 PMC1809430

[185] Sánchez-Muñoz F Fonseca-Camarillo G Villeda-Ramírez MA Miranda-Pérez E Mendivil EJ Barreto-Zúñiga R. Transcript levels of toll-like receptors 5, 8 and 9 correlate with inflammatory activity in ulcerative colitis. BMC Gastroenterol. (2011) 11:1–9. doi: 10.1186/1471-230X-11-138 22185629 PMC3287145

[186] Hall JA Bouladoux N Sun CM Wohlfert EA Blank RB Zhu Q. Commensal DNA limits regulatory T cell conversion and is a natural adjuvant of intestinal immune responses. Immunity. (2008) 29:637–49. doi: 10.1016/j.immuni.2008.08.009, PMID: 18835196 PMC2712925

[187] Hui W Shuhua L Houzhong L Fengxia D Jie G Yanmin W. Mechanism of probiotic VSL# 3 inhibiting NF-κB and TNF-α on colitis through TLR4-NF-κB signal pathway. Iran J Public Health. (2019) 48:1292. 31497551 PMC6708536

[188] Feng Y Cui Y Gao J-L Li M-H Li R Jiang X-H. Resveratrol attenuates neuronal autophagy and inflammatory injury by inhibiting the TLR4/NF-κB signaling pathway in experimental traumatic brain injury. Int J Mol Med. (2016) 37:921–30. doi: 10.3892/ijmm.2016.2495, PMID: 26936125 PMC4790669

[189] He X Wei Z Wang J Kou J Liu W Fu Y. Alpinetin attenuates inflammatory responses by suppressing TLR4 and NLRP3 signaling pathways in DSS-induced acute colitis. Sci Rep. (2016) 6:1–11. doi: 10.1038/srep28370 27321991 PMC4913257

[190] Bain CC Mowat AM. Macrophages in intestinal homeostasis and inflammation. Immunol Rev. (2014) 260:102–17. doi: 10.1111/imr.12192, PMID: 24942685 PMC4141699

[191] Gabanyi I Muller PA Feighery L Oliveira TY Costa-Pinto FA Mucida D. Neuro-immune interactions drive tissue programming in intestinal macrophages. Cell. (2016) 164:378–91. doi: 10.1016/j.cell.2015.12.023, PMID: 26777404 PMC4733406

[192] Sun MC Zhang FC Yin X Cheng BJ Zhao CH Wang YL. *Lactobacillus reuteri* F-9-35 prevents DSS-induced colitis by inhibiting proinflammatory gene expression and restoring the gut microbiota in mice. J Food Sci. (2018) 83:2645–52. doi: 10.1111/1750-3841.14326 30216448

[193] Zhang Z Shen P Liu J Gu C Lu X Li Y. *In vivo* study of the efficacy of the essential oil of Zanthoxylum bungeanum pericarp in dextran sulfate sodium-induced murine experimental colitis. J Agric Food Chem. (2017) 65:3311–9. doi: 10.1021/acs.jafc.7b01323, PMID: 28368613

[194] Chung I-C Ouyang C-N Yuan S-N Lin H-C Huang K-Y Wu P-S. Pretreatment with a heat-killed probiotic modulates the NLRP3 inflammasome and attenuates colitis-associated colorectal cancer in mice. Nutrients. (2019) 11:516. doi: 10.3390/nu11030516, PMID: 30823406 PMC6471765

[195] Dou X Qiao L Chang J Yan S Song X Chen Y. *Lactobacillus casei* ATCC 393 and it's metabolites alleviate dextran sulphate sodium-induced ulcerative colitis in mice through the NLRP3-(Caspase-1)/IL-1β pathway. Food Funct. (2021) 12:12022–35. doi: 10.1039/D1FO02405A 34755743

[196] Xu C Yan S Guo Y Qiao L Ma L Dou X. *Lactobacillus casei* ATCC 393 alleviates Enterotoxigenic *Escherichia coli* K88-induced intestinal barrier dysfunction *via* TLRs/mast cells pathway. Life Sci. (2020) 244:117281. doi: 10.1016/j.lfs.2020.117281, PMID: 31926249

[197] Pittayanon R Lau JT Leontiadis GI Tse F Yuan Y Surette M. Differences in gut microbiota in patients with vs without inflammatory bowel diseases: a systematic review. Gastroenterology. (2020) 158:e931:930–946.e1. doi: 10.1053/j.gastro.2019.11.294 31812509

[198] Li Z-Y Lin L-H Liang H-J Li Y-Q Zhao F-Q Sun T-Y. *Lycium barbarum* polysaccharide alleviates DSS-induced chronic ulcerative colitis by restoring intestinal barrier function and modulating gut microbiota. Ann Med. (2023) 55:2290213. doi: 10.1080/07853890.2023.2290213, PMID: 38061697 PMC10836275

[199] Shang Q Wang Y Pan L Niu Q Li C Jiang H. Dietary polysaccharide from *Enteromorpha clathrata* modulates gut microbiota and promotes the growth of *Akkermansia muciniphila*, Bifidobacterium spp. and Lactobacillus spp. Mar Drugs. (2018) 16:167. doi: 10.3390/md16050167, PMID: 29772753 PMC5983298

[200] Zhao T Zhang Y Nan L Zhu Q Wang S Xie Y. Impact of structurally diverse polysaccharides on colonic mucin O-glycosylation and gut microbiota. npj Biofilms Microb. (2023) 9:97. doi: 10.1038/s41522-023-00468-3 PMC10713555 38081891

[201] Alam A Leoni G Quiros M Wu H Desai C Nishio H. The microenvironment of injured murine gut elicits a local pro-restitutive microbiota. Nat Microbiol. (2016) 1:1–8. doi: 10.1038/nmicrobiol.2015.21 PMC5076466 27571978

[202] Bian X Wu W Yang L Lv L Wang Q Li Y. Administration of *Akkermansia muciniphila* ameliorates dextran sulfate sodium-induced ulcerative colitis in mice. Front Microbiol. (2019) 10:2259. doi: 10.3389/fmicb.2019.02259 31632373 PMC6779789

[203] Ganesh BP Klopfleisch R Loh G Blaut M. Commensal *Akkermansia muciniphila* exacerbates gut inflammation in *Salmonella Typhimurium*-infected gnotobiotic mice. PLoS One. (2013) 8:e74963. doi: 10.1371/journal.pone.0074963, PMID: 24040367 PMC3769299

[204] Seregin SS Golovchenko N Schaf B Chen J Pudlo NA Mitchell J. NLRP6 protects Il10−/− mice from colitis by limiting colonization of *Akkermansia muciniphila*. Cell Rep. (2017) 19:733–45. doi: 10.1016/j.celrep.2017.03.080, PMID: 28445725 PMC5528001

[205] Gómez-Gallego C Pohl S Salminen S De Vos W Kneifel W. *Akkermansia muciniphila*: a novel functional microbe with probiotic properties. Benefic Microbes. (2016) 7:571–84. doi: 10.3920/BM2016.0009, PMID: 27291403

[206] Ansaldo E Slayden LC Ching KL Koch MA Wolf NK Plichta DR. *Akkermansia muciniphila* induces intestinal adaptive immune responses during homeostasis. Science. (2019) 364:1179–84. doi: 10.1126/science.aaw7479, PMID: 31221858 PMC6645389

[207] Itani S Watanabe T Nadatani Y Sugimura N Shimada S Takeda S. NLRP3 inflammasome has a protective effect against oxazolone-induced colitis: a possible role in ulcerative colitis. Sci Rep. (2016) 6:39075. doi: 10.1038/srep39075, PMID: 27966619 PMC5155456

[208] Nowarski R Jackson R Gagliani N De Zoete MR Palm NW Bailis W. Epithelial IL-18 equilibrium controls barrier function in colitis. Cell. (2015) 163:1444–56. doi: 10.1016/j.cell.2015.10.072, PMID: 26638073 PMC4943028

[209] Allen IC Tekippe EM Woodford R-MT Uronis JM Holl EK Rogers AB. The NLRP3 inflammasome functions as a negative regulator of tumorigenesis during colitis-associated cancer. J Exp Med. (2010) 207:1045–56. doi: 10.1084/jem.20100050, PMID: 20385749 PMC2867287

[210] Hirota SA Ng J Lueng A Khajah M Parhar K Li Y. NLRP3 inflammasome plays a key role in the regulation of intestinal homeostasis. Inflamm Bowel Dis. (2011) 17:1359–72. doi: 10.1002/ibd.21478, PMID: 20872834 PMC3026862

[211] Zaki MH Boyd KL Vogel P Kastan MB Lamkanfi M Kanneganti T-D. The NLRP3 inflammasome protects against loss of epithelial integrity and mortality during experimental colitis. Immunity. (2010) 32:379–91. doi: 10.1016/j.immuni.2010.03.003, PMID: 20303296 PMC2982187

[212] Aghamohammad S Sepehr A Miri ST Najafi S Pourshafie MR Rohani M. Anti-inflammatory and immunomodulatory effects of Lactobacillus spp. as a preservative and therapeutic agent for IBD control. Immun Inflamm Dis. (2022) 10:e635. doi: 10.1002/iid3.635, PMID: 35634951 PMC9119005

[213] Farzaei MH El-Senduny FF Momtaz S Parvizi F Iranpanah A Tewari D. An update on dietary consideration in inflammatory bowel disease: anthocyanins and more. Expert Rev Gastroenterol Hepatol. (2018) 12:1007–24. doi: 10.1080/17474124.2018.1513322 30136591

[214] Kasti AN Synodinou KD Pyrousis IA Nikolaki MD Triantafyllou KD. Probiotics regulating inflammation *via* NLRP3 inflammasome modulation: a potential therapeutic approach for COVID-19. Microorganisms. (2021) 9:2376. doi: 10.3390/microorganisms9112376, PMID: 34835501 PMC8624812

[215] Shi H Deng X Deng Q Liu Z Liu N. Probiotic lactobacilli improved growth performance and attenuated *Salmonella Typhimurium* infection *via* jak/stat signaling in broilers. Braz J Poult Sci. (2021) 23. doi: 10.1590/1806-9061-2020-1328

[216] Li Y Yu C Zhu W-M Xie Y Qi X Li N. Triptolide ameliorates IL-10-deficient mice colitis by mechanisms involving suppression of IL-6/STAT3 signaling pathway and down-regulation of IL-17. Mol Immunol. (2010) 47:2467–74. doi: 10.1016/j.molimm.2010.06.007, PMID: 20615550

[217] Rosen MJ Chaturvedi R Washington MK Kuhnhein LA Moore PD Coggeshall SS. STAT6 deficiency ameliorates severity of oxazolone colitis by decreasing expression of claudin-2 and Th2-inducing cytokines. J Immunol. (2013) 190:1849–58. doi: 10.4049/jimmunol.1201373, PMID: 23303670 PMC3563924

[218] Agrawal M Kim ES Colombel J-F. JAK inhibitors safety in ulcerative colitis: practical implications. J Crohn's Colitis. (2020) 14:S755–60. doi: 10.1093/ecco-jcc/jjaa017, PMID: 32006031 PMC7395307

[219] Aghamohammad S Sepehr A Miri ST Najafi S Pourshafie MR Rohani M. The role of combining probiotics in preventing and controlling inflammation: a focus on the anti-inflammatory and immunomodulatory effects of probiotics in an *in vitro* model of IBD. Can J Gastroenterol Hepatol. (2022) 2022:1–9. doi: 10.1155/2022/2045572 PMC9666007 36397951

[220] Rohani M Noohi N Talebi M Katouli M Pourshafie MR. Highly heterogeneous probiotic Lactobacillus species in healthy Iranians with low functional activities. PLoS One. (2015) 10:e0144467. doi: 10.1371/journal.pone.0144467, PMID: 26645292 PMC4672925

[221] Villarino AV Kanno Y O'shea JJ. Mechanisms and consequences of Jak–STAT signaling in the immune system. Nat Immunol. (2017) 18:374–84. doi: 10.1038/ni.3691, PMID: 28323260 PMC11565648

[222] Li W Wang Y Wang C Wang H Ma Y Yang H. Probiotic mixture VSL# 3 prevents ulcerative colitis-associated carcinogenesis in mice and cells by regulating the inflammatory and Wnt/β-catenin pathway. Chin Med J. (2022) 135:2357–9. doi: 10.1097/CM9.0000000000002035, PMID: 35672115 PMC9771233

[223] Nava P Koch S Laukoetter MG Lee WY Kolegraff K Capaldo CT. Interferon-γ regulates intestinal epithelial homeostasis through converging β-catenin signaling pathways. Immunity. (2010) 32:392–402. doi: 10.1016/j.immuni.2010.03.001, PMID: 20303298 PMC2859189

[224] Odegaard JI Ricardo-Gonzalez RR Goforth MH Morel CR Subramanian V Mukundan L. Macrophage-specific PPARγ controls alternative activation and improves insulin resistance. Nature. (2007) 447:1116–20. doi: 10.1038/nature05894, PMID: 17515919 PMC2587297

[225] Park J-S Choi JW Jhun J Kwon JY Lee B-I Yang CW. *Lactobacillus acidophilus* improves intestinal inflammation in an acute colitis mouse model by regulation of Th17 and Treg cell balance and fibrosis development. J Med Food. (2018) 21:215–24. doi: 10.1089/jmf.2017.3990 29336663

[226] Huang J Yang Z Li Y Chai X Liang Y Lin B. *Lactobacillus paracasei* R3 protects against dextran sulfate sodium (DSS)-induced colitis in mice *via* regulating Th17/Treg cell balance. J Transl Med. (2021) 19:1–13. doi: 10.1186/s12967-021-02943-x 34407839 PMC8371868

[227] Hölttä V Klemetti P Salo HM Koivusalo A Pakarinen M Westerholm-Ormio M. Interleukin-17 immunity in pediatric Crohn disease and ulcerative colitis. J Pediatr Gastroenterol Nutr. (2013) 57:287–92. doi: 10.1097/MPG.0b013e3182979252, PMID: 23974060

[228] Miyauchi E Ogita T Miyamoto J Kawamoto S Morita H Ohno H. *Bifidobacterium longum* alleviates dextran sulfate sodium-induced colitis by suppressing IL-17A response: involvement of intestinal epithelial costimulatory molecules. PLoS One. (2013) 8:e79735. doi: 10.1371/journal.pone.0079735, PMID: 24255712 PMC3821848

[229] Ueno A Jijon H Chan R Ford K Hirota C Kaplan GG. Increased prevalence of circulating novel IL-17 secreting Foxp3 expressing CD4+ T cells and defective suppressive function of circulating Foxp3+ regulatory cells support plasticity between Th17 and regulatory T cells in inflammatory bowel disease patients. Inflamm Bowel Dis. (2013) 19:2522–34. doi: 10.1097/MIB.0b013e3182a85709, PMID: 24097227

[230] Jan R-L Yeh K-C Hsieh M-H Lin Y-L Kao H-F Li P-H. *Lactobacillus gasseri* suppresses Th17 pro-inflammatory response and attenuates allergen-induced airway inflammation in a mouse model of allergic asthma. Br J Nutr. (2012) 108:130–9. doi: 10.1017/S0007114511005265, PMID: 21996276

[231] Amdekar S Singh V Kumar A Sharma P Singh R. Lactobacillus casei and *Lactobacillus acidophilus* regulate inflammatory pathway and improve antioxidant status in collagen-induced arthritic rats. J Interf Cytokine Res. (2013) 33:1–8. doi: 10.1089/jir.2012.0034, PMID: 23030670

[232] Chen L-L Zou Y-Y Lu F-G Li F-J Lian G-H. Efficacy profiles for different concentrations of *Lactobacillus acidophilus* in experimental colitis. World J Gastroenterol: WJG. (2013) 19:5347–56. doi: 10.3748/wjg.v19.i32.5347, PMID: 23983440 PMC3752571

[233] Del Zotto B Mumolo G Pronio A Montesani C Tersigni R Boirivant M. TGF-β 1 production in inflammatory bowel disease: differing production patterns in Crohn's disease and ulcerative colitis. Clin Exp Immunol. (2003) 134:120–6. doi: 10.1046/j.1365-2249.2003.02250.x, PMID: 12974764 PMC1808847

[234] Liu Z Yadav PK Xu X Su J Chen C Tang M. The increased expression of IL-23 in inflammatory bowel disease promotes intraepithelial and lamina propria lymphocyte inflammatory responses and cytotoxicity. J Leukoc Biol. (2011) 89:597–606. doi: 10.1189/jlb.0810456, PMID: 21227898

[235] Hart A Lammers K Brigidi P Vitali B Rizzello F Gionchetti P. Modulation of human dendritic cell phenotype and function by probiotic bacteria. Gut. (2004) 53:1602–9. doi: 10.1136/gut.2003.037325, PMID: 15479680 PMC1774301

[236] Owen JL Mohamadzadeh M. Microbial activation of gut dendritic cells and the control of mucosal immunity. J Interf Cytokine Res. (2013) 33:619–31. doi: 10.1089/jir.2013.0046, PMID: 23962004 PMC3814820

[237] Niess JH. Role of mucosal dendritic cells in inflammatory bowel disease. World J Gastroenterol: WJG. (2008) 14:5138–48. doi: 10.3748/wjg.14.5138, PMID: 18777590 PMC2744003

[238] Rescigno M Di Sabatino A. Dendritic cells in intestinal homeostasis and disease. J Clin Invest. (2009) 119:2441–50. doi: 10.1172/JCI39134, PMID: 19729841 PMC2735931

[239] Cătană C-S Neagoe IB Cozma V Magdaş C Tăbăran F Dumitraşcu DL. Contribution of the IL-17/IL-23 axis to the pathogenesis of inflammatory bowel disease. World J Gastroenterol: WJG. (2015) 21:5823–30. doi: 10.3748/wjg.v21.i19.5823, PMID: 26019446 PMC4438016

[240] Mcgovern D Powrie F. The IL23 axis plays a key role in the pathogenesis of IBD. Gut. (2007) 56:1333–6. doi: 10.1136/gut.2006.115402, PMID: 17872562 PMC2000291

[241] Bates J Diehl L. Dendritic cells in IBD pathogenesis: an area of therapeutic opportunity? J Pathol. (2014) 232:112–20. doi: 10.1002/path.4277, PMID: 24122796 PMC4285849

[242] Bsat M Chapuy L Rubio M Wassef R Richard C Schwenter F. Differential pathogenic Th17 profile in mesenteric lymph nodes of Crohn's disease and ulcerative colitis patients. Front Immunol. (2019) 10:1177. doi: 10.3389/fimmu.2019.01177 31191543 PMC6547831

[243] Caprioli F Pallone F Monteleone G. Th17 immune response in IBD: a new pathogenic mechanism. J Crohn's Colitis. (2008) 2:291–5. doi: 10.1016/j.crohns.2008.05.004, PMID: 21172226

[244] Hempel S Newberry S Ruelaz A Wang Z Miles J Suttorp MJ. Safety of probiotics used to reduce risk and prevent or treat disease. Evid Rep Technol Assess. (2011):1–645. PMID: 23126627 PMC4780970

[245] Zeng J Li YQ Zuo XL Zhen YB Yang J Liu CH. Clinical trial: effect of active lactic acid bacteria on mucosal barrier function in patients with diarrhoea-predominant irritable bowel syndrome. Aliment Pharmacol Ther. (2008) 28:994–1002. doi: 10.1111/j.1365-2036.2008.03818.x 18671775

[246] Doron S Snydman DR. Risk and safety of probiotics. Clin Infect Dis. (2015) 60:S129–34. doi: 10.1093/cid/civ085, PMID: 25922398 PMC4490230

[247] Sanders ME Akkermans LM Haller D Hammerman C Heimbach JT Hörmannsperger G. Safety assessment of probiotics for human use. Gut Microbes. (2010) 1:164–85. doi: 10.4161/gmic.1.3.12127, PMID: 21327023 PMC3023597

[248] Healey GR Murphy R Brough L Butts CA Coad J. Interindividual variability in gut microbiota and host response to dietary interventions. Nutr Rev. (2017) 75:1059–80. doi: 10.1093/nutrit/nux062, PMID: 29190368

[249] Sang L-X Chang B Dai C Gao N Liu W-X Jiang M. Heat-killed VSL# 3 ameliorates dextran sulfate sodium (DSS)-induced acute experimental colitis in rats. Int J Mol Sci. (2013) 15:15–28. doi: 10.3390/ijms15010015, PMID: 24451125 PMC3907795

[250] Zhang M Qiu X Zhang H Yang X Hong N Yang Y. *Faecalibacterium prausnitzii* inhibits interleukin-17 to ameliorate colorectal colitis in rats. PLoS One. (2014) 9:e109146. doi: 10.1371/journal.pone.0109146, PMID: 25275569 PMC4183556

[251] Sang L-X Chang B Wang B-Y Liu W-X Jiang M. Live and heat-killed probiotic: effects on chronic experimental colitis induced by dextran sulfate sodium (DSS) in rats. Int J Clin Exp Med. (2015) 8:20072–8. PMID: 26884919 PMC4723764

[252] Lamubol J Ohto N Kuwahara H Mizuno M. *Lactiplantibacillus plantarum* 22A-3-induced TGF-β1 secretion from intestinal epithelial cells stimulated CD103+ DC and Foxp3+ Treg differentiation and amelioration of colitis in mice. Food Funct. (2021) 12:8044–55. doi: 10.1039/D1FO00990G 10.1039/d1fo00990g 34282811

